# Supplementary material for: 2H-Thiopyran-2-thione sulfine, a compound for converting H2S to HSOH/H2S2 and increasing intracellular sulfane sulfur levels
Source: Nat Commun. 2024 Mar 19;15:2453. doi: 10.1038/s41467-024-46652-7 (PMC10951338; doi:10.1038/s41467-024-46652-7)
Supplement: Supplementary file 1 — Supplementary Information [file 41467_2024_46652_MOESM1_ESM.pdf]

Supplementary Information

2*H*-Thiopyran-2-Thione Sulfine, a Compound For Converting H_2S to HSOH/H_2S_2 and Increasing Intracellular Sulfane Sulfur Levels

Qi Cui,[1,‡] Meg Shieh,[1,‡] Tony W. Pan,[1] Akiyuki Nishimura,[2] Tetsuro Matsunaga,[3] Shane S. Kelly,[4] Shi Xu,[1] Minkyung Jung,[3] Seiryo Ogata,[3] Masanobu Morita,[3] Jun Yoshitake,[3] Xiaoyan Chen,[1] Jerome R. Robinson,[1] Wei-Jun Qian,[4] Motohiro Nishida,[2,5] Takaaki Akaike,[3,*] and Ming Xian[1,*]

[1] Department of Chemistry, Brown University, Providence, Rhode Island 02912, United States

[2] Division of Cardiocirculatory Signaling, National Institute for Physiological Sciences (NIPS) and Exploratory Research Center on Life and Living Systems (ExCELLS), National Institutes of Natural Sciences, Okazaki 444-8787, Japan

[3] Department of Environmental Medicine and Molecular Toxicology, Tohoku University Graduate School of Medicine, Sendai 980-8575, Japan

[4] Biological Sciences Division, Pacific Northwest National Laboratory, Richland, Washington 99352, United States

[5] Department of Physiology, Graduate School of Pharmaceutical Sciences, Kyushu University, Fukuoka 812-8582, Japan

[‡]These authors contributed equally: Qi Cui, Meg Shieh.

Supplementary Methods

All the reactions were stirred using Teflon-coated magnetic stir bars. Elevated temperatures were maintained using Thermostat-controlled silicone oil baths. Chemicals were purchased from Aldrich, Alfa Aesar, TCI, and used without further purification, unless otherwise indicated. Analytical TLC was performed with 0.25 mm silica gel G plates with a 254 nm fluorescent indicator. The TLC plates were visualized by ultraviolet light and treatment with anisaldehyde-H_2SO_4 stain followed by gentle heating. Purification of products was accomplished by flash chromatography on silica gel and the purified compounds show a single spot by analytical TLC.

NMR spectra were measured on Bruker high field NMR spectrometers (^1H at 400MHz or 600 MHz, ^{13}C at 101 MHz or 151 MHz) nuclear magnetic resonance spectrometers. Data for ^1H-NMR spectra are reported as follows: chemical shift (ppm, referenced to residual solvent peak $CDCl_3$: 7.26 ppm, CD_2Cl_2: 5.32 ppm, CD_3OD: 3.31 ppm); s = singlet, d = doublet, t = triplet, q = quartet, dd = doublet of doublets, m = multiplet), coupling constant (Hz), and integration. Data for ^{13}C-NMR are reported in terms of chemical shift (ppm) relative to residual solvent peak ($CDCl_3$: 77.16 ppm, CD_2Cl_2: 53.84 ppm, CD_3OD: 49.00 ppm). High-resolution mass spectra (HRMS) were recorded on the Agilent 6530 LC-MS (ESI). Absorption spectra were recorded on a Thermo Scientific Evolution 300 UV-Vis Spectrophotometer using 1 cm quartz cells. Fluorescence excitation and emission spectra were measured on a Cary Eclipse fluorescence spectrophotometer. Cell imaging was performed using the Keyence All-in-One Fluorescence Microscope (BZ-X810).

Syntheses of substrates:

Compound TT is a known compound and it was prepared following a reported procedure.[1]

TT: ^1H NMR (400 MHz, $CDCl_3$) δ 8.48 (dd, J = 1.8, 1.2 Hz, 1H), 7.58 (dd, J = 10.4, 1.8 Hz, 1H), 7.44 (dd, J = 10.4, 1.2 Hz, 1H), 3.91 (s, 3H).

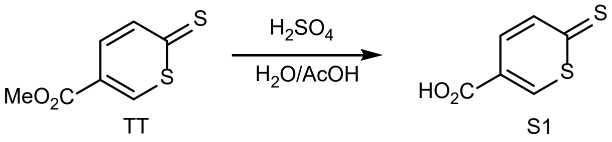

S1: To a stirred solution of methyl 2-thioxo-2*H*-thiopyran-5-carboxylate TT (117 mg, 0.63

mmol) in acetic acid (5 mL), H_2SO_4 (0.75 mL, 50% v/v) was added. After being stirred at 100 °C for 5 h, the reaction was quenched by H_2O and extracted with DCM. Combined organic layers were washed by brine and dried by Na_2SO_4. The solvent was removed by rotary evaporator. The crude material was purified by flash column chromatography (EA with 1% AcOH) to yield S1 (84 mg, yield = 78 %) as a red powder.

S1: R_f: 0.05 (EA), m.p. > 200 °C. ^{1}H NMR (600 MHz, CD_3OD) δ 8.72 (dd, J = 1.9, 1.1 Hz, 1H), 7.67 (dd, J = 10.3, 1.9 Hz, 1H), 7.46 (dd, J = 10.3, 1.1 Hz, 1H). ^{13}C NMR (151 MHz, CD_3OD) δ 207.8, 165.3, 150.1, 140.3, 133.0, 127.3. HRMS (ESI, *m/z*): $[M+H]^+$ calculated for $C_6H_5O_2S_2^+$: 172.9731; found: 172.9718.

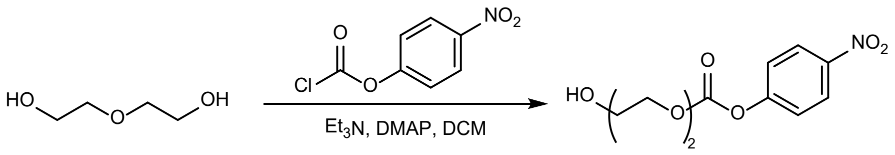

S2

S2: To a stirred solution of diethylene glycol (1.92 mL, 20 mmol, 2.0 eq) in DCM (70 mL), a mixture of 4-nitrophenyl carbonochloridate (2.02 g, 10 mmol, 1.0 eq), Et_3N (2.76 mL, 20 mmol, 2.0 eq), DMAP (61 mg, 0.5 mmol, cat.) and DCM (30 mL) was added dropwise at 0 °C. The mixture was stirred at room temperature for 30 min and then concentrated under reduced pressure. The residue was diluted with H_2O and extracted with EA. Combined organic layers were washed by brine and dried by Na_2SO_4. The solvent was removed by rotary evaporator. The crude material was purified by flash column chromatography (HEX/EA = 1/1–1/2) to yield S2 (365 mg, yield = 13 %) as a colorless oil.

S2: R_f: 0.2 (EA). ^{1}H NMR (600 MHz, $CDCl_3$) δ 8.28 (d, J = 8.7 Hz, 2H), 7.39 (d, J = 8.7 Hz, 2H), 4.46 (t, J = 4.5 Hz, 2H), 3.82 (t, J = 4.5 Hz, 2H), 3.78 (t, J = 4.5 Hz, 2H), 3.66 (t, J = 4.5 Hz, 2H), 1.80 (brs, 1H). ^{13}C NMR (151 MHz, $CDCl_3$) δ 155.6, 152.7, 145.6, 125.5, 121.9, 72.6, 68.8, 68.3, 61.9. HRMS (ESI, *m/z*): $[M+H]^+$ calculated for $C_{11}H_{14}NO_4^+$: 272.0770; found: 272.0766.

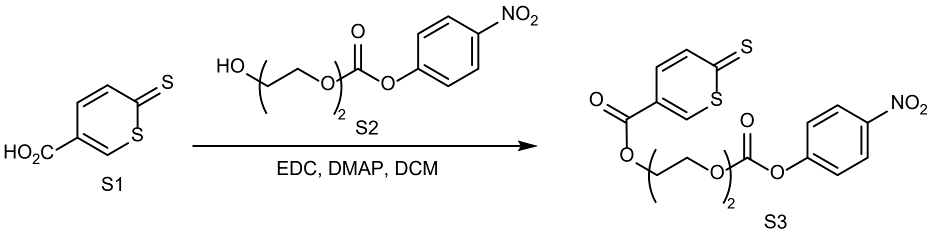

S3: To a stirred solution of S1 (181 mg, 1.05 mmol, 1.05 eq), S2 (271 mg, 1.0 mmol, 1.0 eq), DMAP (12 mg, 0.1 mmol, 0.1 eq) in DCM (10 mL), EDC (288 mg, 1.5 mmol, 1.5 eq) was added. The mixture was stirred at room temperature for 24 h under Ar atmosphere. Then, the reaction was quenched by H_2O and extracted by EA. Combined organic layers were washed by brine and dried by Na_2SO_4. The solvent was removed by rotary evaporator. The crude material was purified by flash column chromatography (HEX/EA = 3/1–2/1) to yield S3 (180.5 mg, yield = 42 %) as a red powder, which was used directly for next step.

TAGDD-6 is a known compound and was prepared using the reported procedure.[2]

TAGDD-6: ^{1}H NMR (400 MHz, $CDCl_3$) δ 7.18 (t, J = 9.0 Hz, 1H), 6.99 (d, J = 9.0 Hz, 1H), 6.94 – 6.86 (m, 1H), 6.75 – 6.70 (m, 1H), 6.19 (s, 1H), 2.32 (s, 6H).

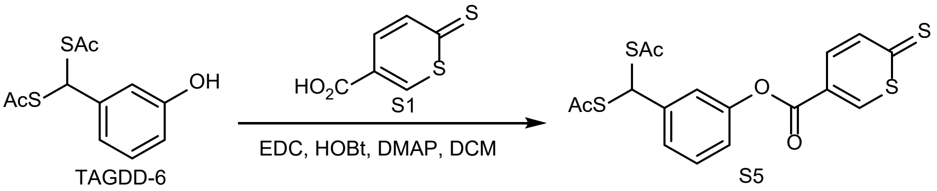

S5: To a stirred solution of TAGDD-6 (166 mg, 0.65 mmol, 1.05 eq), S1 (107 mg, 0.62 mmol, 1.0 eq), DMAP (17 mg, 0.14 mmol, 0.2 eq), HOBt (142 mg, 0.93 mmol, 1.5 eq) in DCM (15 mL), EDC (178 mg, 0.93 mmol, 1.5 eq) was added. After being stirred at room temperature for 24 h under Ar atmosphere, the reaction was quenched by H_2O and extracted by EA. Combined organic layers were washed by brine and dried by Na_2SO_4. The solvent was removed by rotary evaporator. The crude material was purified by flash column chromatography (HEX/EA = 5/1–3/1) to yield S5 (163 mg, yield = 64 %) as a red powder, which was used directly for next step.

X-ray Crystallography:

Crystals of TTS-Br were removed from the mother liquor and transferred to a glass slide covered in Paraton™ oil where it was evaluated and mounted with the assistance of an optical

microscope. X-ray reflection intensity data were collected on a Bruker D8 Venture with a Photon III CPAD detector employing graphite-monochromated Mo-Kα radiation (λ = 0.71073 Å) at a temperature of 173(1) K. Rotation frames were integrated using SAINT,[3] producing a listing of unaveraged F^2 and $\sigma(F^2)$ values which were then passed to the SHELXT[4] program package for further processing and structure solution. The intensity data were corrected for Lorentz and polarization effects and for absorption using SADABS.[5] The structures were solved by direct methods (SHELXT).[4] Refinement was by full-matrix least squares based on F^2 using SHELXL.[6] All reflections were used during refinements. Non-hydrogen atoms were refined anisotropically and hydrogen atoms were refined using a riding model. The data is shown in Supplementary Tables 1-4.

Supplementary Table 1. Crystal data and structure refinement for TTS-Br.

Identification code	mo_MX20230412_0m
Empirical formula	$C_{12}H_7BrO_3S_2$
Formula weight	343.21
Temperature/K	173
Crystal system	orthorhombic
Space group	$P2_12_12_1$
a/Å	3.8876(5)
b/Å	11.7482(12)
c/Å	27.410(3)
α/°	90
β/°	90
γ/°	90
Volume/Å^3	1251.9(3)
Z	4
ρ_{calc}g/cm^3	1.821
μ/mm^{-1}	3.612
F(000)	680.0
Crystal size/mm^3	0.1 × 0.03 × 0.01
Radiation	MoKα (λ = 0.71073)
2Θ range for data collection/°	4.566 to 54.998
Index ranges	$-5 \le h \le 5$, $-15 \le k \le 15$, $-35 \le l \le 35$
Reflections collected	43660
Independent reflections	2838 [R_{int} = 0.0461, R_{sigma} = 0.0240]
Data/restraints/parameters	2838/0/164
Goodness-of-fit on F^2	1.206

Final R indexes [I>=2σ (I)]	R_1 = 0.0466, wR_2 = 0.1307
Final R indexes [all data]	R_1 = 0.0479, wR_2 = 0.1316
Largest diff. peak/hole / e Å^{-3}	1.15/-0.59
Flack parameter	0.06(2)
CCDC Deposit #	2259484

Supplementary Table 2. Bond distances for TTS-Br.

Atom	Atom	Length (Å)	Atom	Atom	Length (Å)
Br1	C10	1.903(7)	C3	C4	1.432(10)
S1	O1	1.381(10)	C4	C5	1.339(10)
S1	C1	1.670(8)	C4	C6	1.482(10)
S2	C1	1.722(10)	C7	C8	1.367(10)
S2	C5	1.702(8)	C7	C12	1.381(9)
O2	C6	1.192(9)	C8	C9	1.387(11)
O3	C6	1.348(8)	C9	C10	1.385(10)
O3	C7	1.403(8)	C10	C11	1.375(10)
C1	C2	1.481(12)	C11	C12	1.394(10)
C2	C3	1.448(10)			

Supplementary Table 3. Bond angles for TTS-Br.

Atom	Atom	Atom	Angle (˚)	Atom	Atom	Atom	Angle (˚)
O1	S1	C1	109.6(5)	O2	C6	C4	124.3(7)
C5	S2	C1	101.5(4)	O3	C6	C4	111.6(6)
C6	O3	C7	118.5(5)	C8	C7	O3	116.4(6)
S1	C1	S2	115.4(5)	C8	C7	C12	122.1(7)
C2	C1	S1	116.3(7)	C12	C7	O3	121.4(6)
C2	C1	S2	128.2(6)	C7	C8	C9	119.7(6)
C3	C2	C1	114.1(7)	C10	C9	C8	118.6(7)
C4	C3	C2	126.6(7)	C9	C10	Br1	119.5(5)
C3	C4	C6	116.5(6)	C11	C10	Br1	118.7(5)
C5	C4	C3	122.8(7)	C11	C10	C9	121.8(6)
C5	C4	C6	120.7(6)	C10	C11	C12	119.4(6)
C4	C5	S2	126.8(6)	C7	C12	C11	118.4(6)
O2	C6	O3	124.0(7)				

Supplementary Table 4. Torsion angles for TTS-Br.

A	B	C	D	Angle (˚)	A	B	C	D	Angle (˚)
Br1	C10	C11	C12	178.7(5)	C5	S2	C1	C2	-1.8(8)
S1	C1	C2	C3	-178.6(6)	C5	C4	C6	O2	169.1(8)
S2	C1	C2	C3	2.4(11)	C5	C4	C6	O3	-9.2(10)
O1	S1	C1	S2	0.1(7)	C6	O3	C7	C8	-122.8(7)

A	B	C	D	Angle (˚)	A	B	C	D	Angle (˚)
O1	S1	C1	C2	-179.1(6)	C6	O3	C7	C12	60.5(9)
O3	C7	C8	C9	-178.0(7)	C6	C4	C5	S2	-174.7(6)
O3	C7	C12	C11	177.4(6)	C7	O3	C6	O2	-3.0(11)
C1	S2	C5	C4	-0.5(8)	C7	O3	C6	C4	175.4(6)
C1	C2	C3	C4	-0.7(12)	C7	C8	C9	C10	1.5(11)
C2	C3	C4	C5	-1.5(13)	C8	C7	C12	C11	0.8(11)
C2	C3	C4	C6	175.4(8)	C8	C9	C10	Br1	-179.1(6)
C3	C4	C5	S2	2.1(12)	C8	C9	C10	C11	-1.2(11)
C3	C4	C6	O2	-7.9(12)	C9	C10	C11	C12	0.7(11)
C3	C4	C6	O3	173.8(7)	C10	C11	C12	C7	-0.5(11)
C5	S2	C1	S1	179.2(5)	C12	C7	C8	C9	-1.3(12)

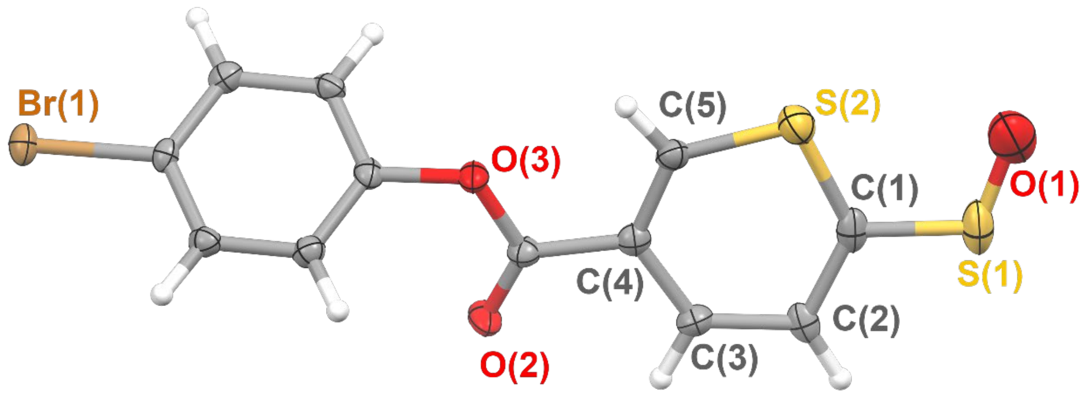

Supplementary Figure 1. Thermal ellipsoid plot (50% probability) of TTS-Br. Selected bond distances, angles, and torsion angles: S(1)–O(1): 1.381(10) Å, S(1)–C(1): 1.670(8) Å, C(1)–C(2): 1.481(12) Å C(2)–C(3): 1.448(10), C(3)–C(4): 1.432(10), C(4)–C(5): 1.339(10), C(5)–S(2): 1.702(8), S(2)–C(1): 1.722(10) Å. S(2)–C(1)–S(1): 115.4(5)˚. C(1)–S(1)–O(1): 109.6(5)˚. S(2)–C(1)–S(1)–O(1): 0.1(7)˚, C(1)–C(2)–C(3)–C(4): -0.7(12)˚, C(1)–S(2)–C(5)–C(4): -0.5(8)˚

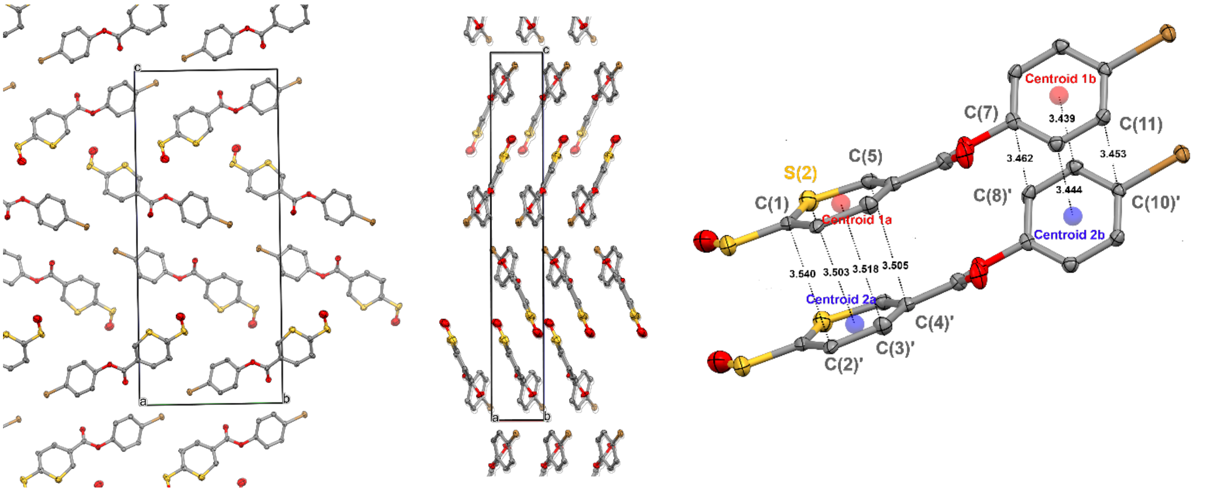

Supplementary Figure 2. Packing diagram of TTS-Br. Interlayer, π-π stacking distance is ~3.44 – 3.52 Å.

Experiments for mechanism studies:

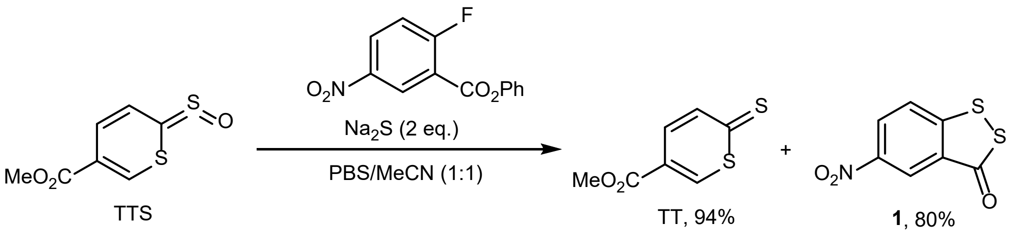

To a stirred solution of TTS (19.6 mg, 0.1 mmol) in MeCN (5 mL), $Na_2S\cdot 9H_2O$ (48 mg, 0.2 mmol, 2.0 eq) in PBS buffer (5X pH = 7.4, 6 mL) was added dropwise. After being stirred at room temperature for 1 h, phenyl 2-fluoro-5-nitrobenzoate (52 mg, 0.2 mmol, in 1 mL MeCN) was added dropwise. The reaction was stirred for another 1 h and then quenched by H_2O and extracted by EA. Combined organic layers were washed by brine and dried by Na_2SO_4. The solvent was removed by rotary evaporator. The crude material was purified by Perp TLC (HEX/EA = 5/1) to yield TT (17.0 mg, yield = 94 %) and **1** (16.5 mg, yield = 80%). The ^{1}H-NMR of the products were consistent with reported literatures.

TT: ^{1}H NMR (400 MHz, $CDCl_3$) δ 8.49 (dd, J = 1.9, 1.1 Hz, 1H), 7.61 (dd, J = 10.3, 1.9 Hz, 1H), 7.44 (dd, J = 10.3, 1.1 Hz, 1H), 3.91 (s, 3H).

1: ^{1}H NMR (600 MHz, $CDCl_3$) δ 8.79 (d, J = 2.3 Hz, 1H), 8.49 (dd, J = 8.9, 2.3 Hz, 1H), 7.78 (d, J = 8.9 Hz, 1H).

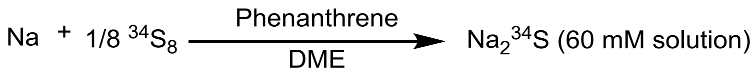

To a stirred suspension of $^{34}S_8$ (31 mg, 0.91 mmol), Phenanthrene (102 mg, 0.57 mmol) in dry DME (5 mL), sodium metal (112 mg, 4.87 mmol) was added under Ar atmosphere. The solution turned dark green when being heated. After being stirred at 80 °C for 16 h, excess sodium metal and the solvent were removed. The residue was partially dissolved in H_2O. The undissolved organic compound was removed by filtration. The concentration of $Na_2{}^{34}S$ solution was analyzed by methylene blue method compared with standard Na_2S sample (~60 mM).

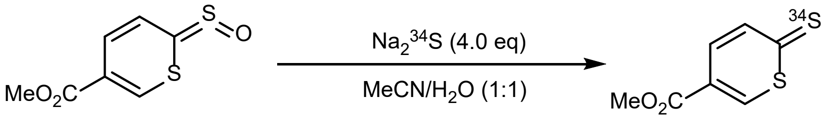

In a 20 mL vial, TTS (19.7 mg, 0.1 mmol, in 6.7 mL MeCN), and $Na_2{}^{34}S$ solution (60 mM in H_2O, 6.7 mL, 0.4 mmol) were added. The reaction was incubated at room temperature for 30 min, which was monitored by UV-Vis spectroscopy. The reaction was quenched by H_2O and extracted by EA. Combined organic layers were washed by brine and dried by Na_2SO_4. The solvent was removed by rotary evaporator. The residue was purified by flash column chromatography (HEX/EA = 5/1) to yield TT-^{34}S (9.5 mg, yield = 52%) as a dark red solid.

TT-^{34}S HRMS (ESI, *m/z*): $[M+H]^+$ calculated for $C_7H_7O_2S^{34}S^+$: 188.9840; found: 188.9866.

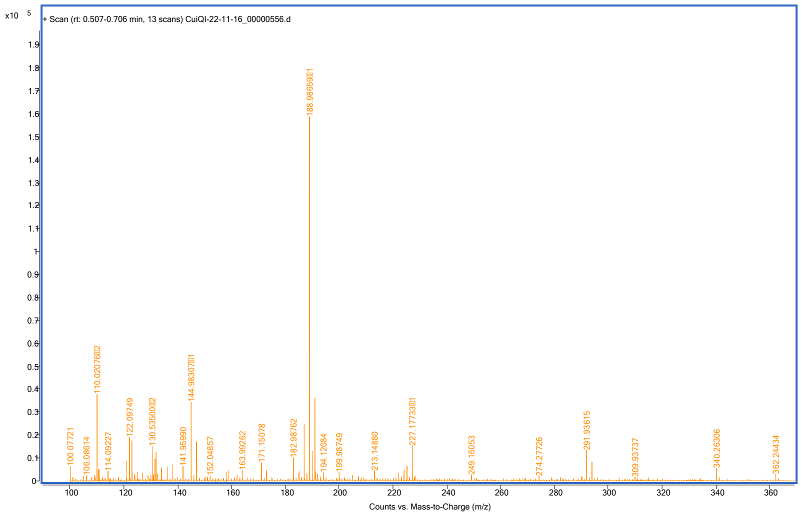

Supplementary Figure 3. TT-^{34}S HRMS spectrum

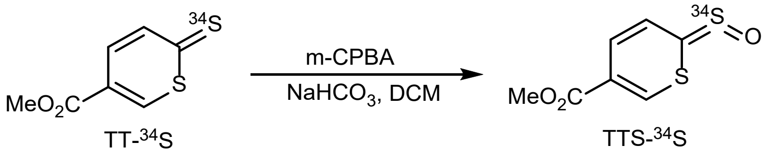

To a stirred solution of TT-^{34}S (6.2 mg, 0.03 mmol) in DCM (1 mL), $NaHCO_3$ (13 mg, 0.15 mmol, 5.0 eq) and *m*-CPBA (70%, 7.4 mg, 0.03 mmol, 1.0 eq) were added. After being stirred at room temperature for 30 min, the reaction was quenched by sat. $NaHCO_3$ solution and extracted by EA. Combined organic layers were washed by brine and dried by Na_2SO_4. The solvent was removed by rotary evaporator. The residue was purified by flash column chromatography (HEX/EA = 5/1–3/1) to yield TTS-^{34}S (4.8 mg, yield = 71 %) as a dark red powder.

TTS-^{34}S HRMS (ESI, *m/z*): $[M+H]^+$ calculated for $C_7H_7O_3S^{34}S^+$: 204.9798; found: 204.9802.

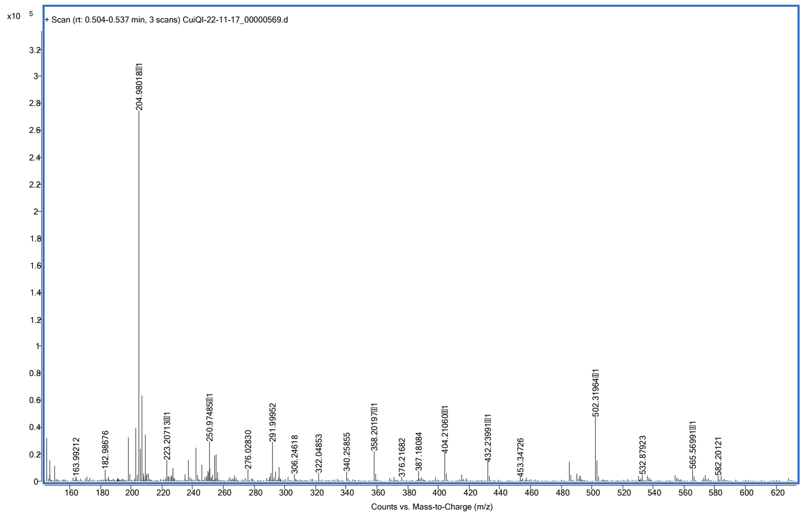

Supplementary Figure 4. TTS-^{34}S HRMS spectrum

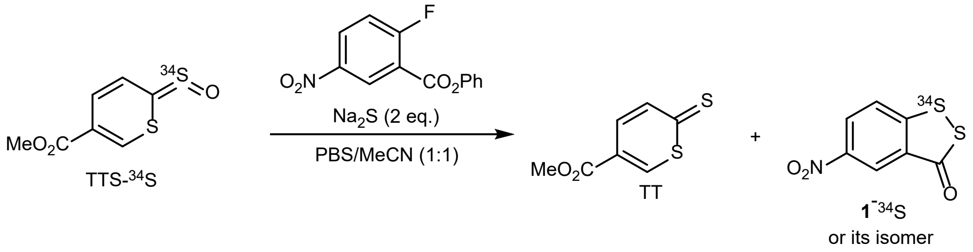

To a solution of TTS-^{34}S (4.1 mg, 0.02 mmol) in MeCN (2 mL), Na_2S solution (2.0 mL, 20 mM in PBS buffer) was added dropwise. After being incubated at room temperature for 10 min, phenyl 2-fluoro-5-nitrobenzoate (5.2 mg, 0.02 mmol) was added. The reaction was incubated

for another 1 h. TT and **1-^{34}S** were detected by LC-MS.

TT**:** HRMS (ESI, *m/z*): $[M+H]^+$ calculated for $C_7H_7O_2S_2^+$: 186.9882; found: 186.9891.

1-^{34}S: HRMS (ESI, *m/z*): $[M+H]^+$ calculated for $C_7H_4NO_3{}^{34}SS^+$: 215.9585; found: 215.9604.

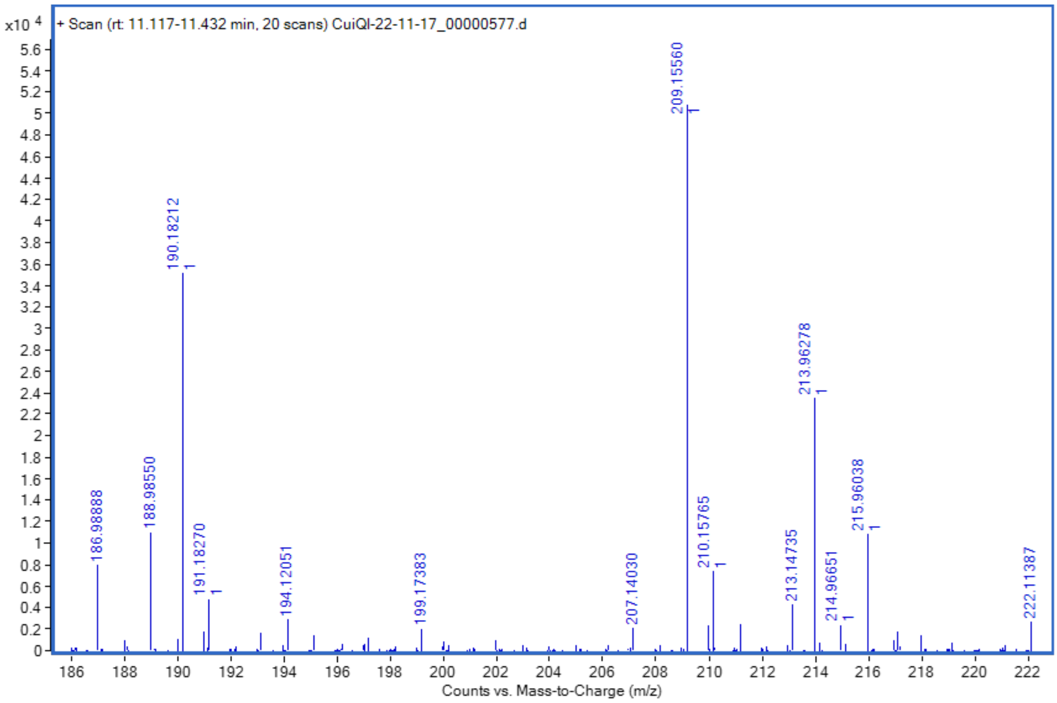

Supplementary Figure 5. HRMS spectrum of **1-**^{34}S.

Kinetic study of the reactions between TT and ROS:

Stock solutions of ROS were freshly made with deionized water and used immediately.

H_2O_2 was diluted from a commercially available solution (30%). The concentration of H_2O_2 was determined using the absorption at 240 nm ($\varepsilon = 43.6\ M^{-1}\ cm^{-1}$).

^{t}BuOOH was diluted from commercially available solutions (70%).

AcOOH was diluted from commercially available solutions (32%).

NaOCl was diluted from commercially available solution with 11-15% available chlorine. The concentration of [$^-$OCl] was determined using the absorption at 292 nm ($\varepsilon = 391\ M^{-1}\ cm^{-1}$).

TT was dissolved in DMSO to afford a 10 mM stock solution.

For H_2O_2, ^{t}BuOOH and AcOOH, TT (10 μM) and ROS (1 mM) were mixed in PBS buffer, pH = 7.4 with DMSO (1%) under room temperature. For NaClO, TT (100 μM) and NaClO (1 mM)

were mixed in PBS buffer, pH = 7.4 with DMSO (1%) under room temperature. UV-Vis absorbance at λ = 339 nm was measured periodically, and kinetics were calculated using the method described below.

To determine [TT] at each time point:

$A = \varepsilon_{TTS} \times [TTS] + \varepsilon_{TT} \times [TT]$

Considering the starting concentration of [TT] was 10 μM or 100 μM, [TTS] + [TT] = 10 or 100 μM, let the starting concentration of [TT] = x.

$A = \varepsilon_{TTS} \times (x - [TT]) + \varepsilon_{TT} \times ([TT])$

$\varepsilon_{TTS(339\ nm)} = 0.0029\ \mu M^{-1}\ cm^{-1}$ and $\varepsilon_{TT(339\ nm)} = 0.0142\ \mu M^{-1}\ cm^{-1}$

$$\frac{A - 0.0029x}{0.0113} = [TT]$$

$$k_{obs} = k[ROS], \text{where } [ROS] = 0.001\ M$$

$$k_{obs}t = -\ln\frac{[TT]}{[TT]_0}, \text{where } [TT]_0 = 10 \text{ or } 100\ \mu M$$

In situ NMR study of the reaction between TT and H_2O_2

To an NMR tube, the solution of TT (0.02 mmol, final concentration is 20 mM) in CD_3CN/D_2O (1:1) was added, followed by adding H_2O_2 (10 M, 20 μL). The NMR tube was shaken immediately and incubated at room temperature for 0 min, 30 min, 3 h, and 24 h. The NMR spectrum showed the reaction occurred within 3 h and completed in 24 h. The transformation from TT to TTS was clean.

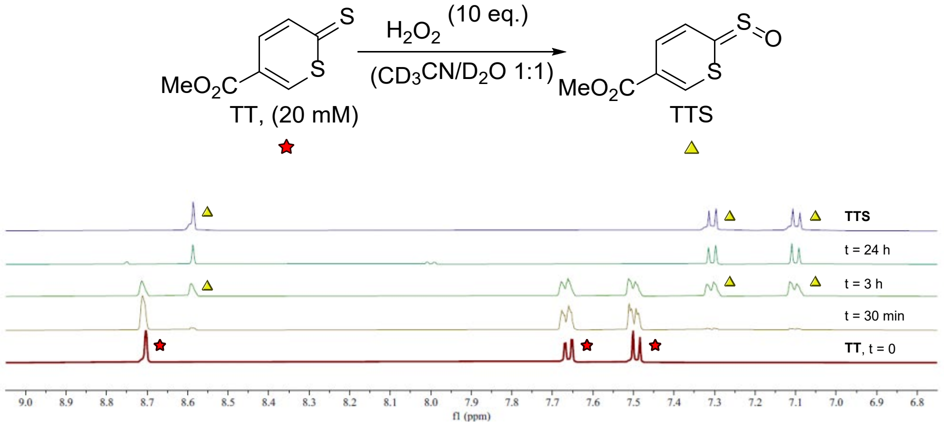

Supplementary Figure 6. ^{1}H NMR spectra of the reaction between TT and H_2O_2 to generate

TTS.

UV-Vis study of TTS and H_2S: TTS (20 μL, 10 mM in DMSO solution, final concentration = 100 μM) and Na_2S (40 μL, 10 mM in H_2O, final concentration = 200 μM) were reacted in PBS buffer, pH = 7.4 (2 mL) at room temperature. The data was collected by UV-Vis spectrometer (700 – 250 nm, every 1 min). There was a significant change in the maximum absorbance wavelength (from 380 nm to 332 nm).

Kinetic study of the reaction between TTS and H_2S: TTS (10 μL, 10 mM in DMSO solution, final concentration = 50 μM) and Na_2S (20 μL, 500 mM in MilliQ H_2O, final concentration = 5 mM), reacted in PBS buffer (50 mM, pH = 7.4) (2 mL) at room temperature. The kinetics were detected by UV-Vis absorbance spectrum (λ = 380 nm) based on the decrease of TTS. The representative kinetic results are shown in Figure S7. We assumed this reaction can be seen as a *pseudo*-first order reaction; the second-order *k* constant (*k*) was calculated by:

$$k_{obs} = k[\mathrm{Na_2S}], \text{where } [\mathrm{Na_2S}] = 0.005\ \mathrm{M}$$

$$k_{obs}t = -\ln\frac{[\mathrm{TTS}]}{[\mathrm{TTS}]_0}, \text{where } [\mathrm{TTS}]_0 = 50\ \mu\mathrm{M}$$

$$k = 2.76\ \mathrm{M^{-1}s^{-1}}$$

The final *k* reported above was calculated by taking the average of the *k* values from 3 trials.

<u>To determine [TTS] at each time point:</u>

$$A = \varepsilon_{TTS} \times [TTS] + \varepsilon_{TT} \times [TT]$$

Considering [TTS] + [TT] = 50 μM,

$$A = \varepsilon_{TTS} \times [TTS] + \varepsilon_{TT} \times (50-[TTS])$$

$\varepsilon_{TTS(380\ nm)} = 0.0147\ \mu M^{-1}\ cm^{-1}$ and $\varepsilon_{TT(380\ nm)} = 0.0025\ \mu M^{-1}\ cm^{-1}$

$$\frac{A - 0.125}{0.0122} = [\mathrm{TTS}]$$

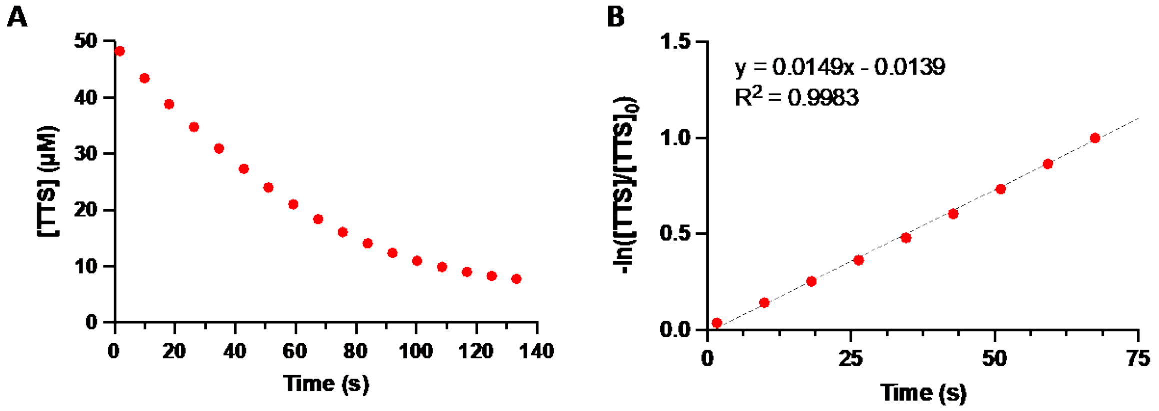

Supplementary Figure 7. Representative time-dependent (A) [TTS] and (B) –ln([TTS]/[TTS]$_0$) changes based on UV-Vis absorption changes at 380 nm of the reaction between TTS (50 μM) and Na_2S (5 mM). Source data are provided as a Source Data file.

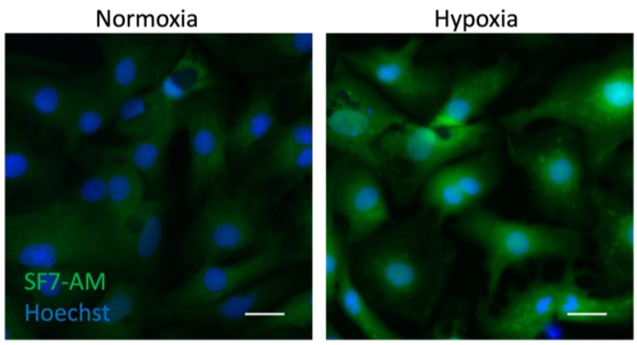

Supplementary Figure 8. Representative fluorescence images of H_2S levels in cells. Neonatal rat cardiomyocytes were placed under normoxic or hypoxic (1% O_2) conditions for 18 h and then incubated with SF7-AM (2.5 μM) and Hoechst (2 μg/mL) for 30 min. Cells were washed three times with HBSS before imaging. Scale bars, 20 μm.

Fluorescence analysis of H_2S_2 in the reaction between Lyso-TTS and H_2S: In the Lyso-TTS/Na_2S experiment, 50 μL of the reaction solution (after Lyso-TTS and Na_2S were mixed and incubated for 30 min) was taken and treated with SSP4 to demonstrate the formation of H_2S_2 in this process. The results showed ~30-fold fluorescence increase, which indicates significant H_2S_2 release in the reaction between Lyso-TTS and H_2S.

Computed energies of all the stationary points:

Thermal correction to Enthalpy *TCH*, Thermal correction to Gibbs Free Energy *TCG*, Sum of electronic and thermal Enthalpies ***H***, Sum of electronic and thermal Free Energies ***G**, delta Gibbs free Energies* ***ΔG***.

Supplementary Table 5: SMD(H_2O)/M06-2X/maug-cc-pVTZ level.

	TCH	***TCG***	***H***	***G***	***ΔG***
**TT**	0.13499	0.083573	-1292.240833	-1292.292251	**0.0**
**HS-**	0.009418	-0.011729	-398.913171	-398.934318	
**TS1**	0.144306	0.087151	-1691.142209	-1691.199364	**15.2**
**INT1**	0.145783	0.088781	-1691.15826	-1691.215263	**5.2**
**INT2**	0.147722	0.091949	-1691.173984	-1691.229757	**-3.9**
**TS2**	0.147159	0.089442	-1691.153769	-1691.211486	**7.6**
**TTS**	0.130347	0.081581	-1217.044028	-1217.092794	**-30.5**
**HOS-**	0.016793	-0.010033	-474.109285	-474.136111	**-3.4**
**HSS-**	0.013154	-0.015325	-797.1089	-797.13738	
**H_2S**	0.018888	-0.005113	-399.369642	-399.393643	
**H_2O**	0.025012	0.002932	-76.41664	-76.43872	

Persulfide metabolome analysis of MEFs treated with TTS: MEFs underwent sonication for homogenization in 0.15 ml of cold methanol solution containing 5 mM β-(4-hydroxyphenyl)ethyl iodoacetamide (HPE-IAM) and 20 mM sodium acetate buffer (pH 6.5), after which the samples were incubated for 20 min at 37 ºC. Following centrifugation (14,000 g for 10 min at 4 ºC), supernatants from the lysates were diluted with 0.1% formic acid containing known amounts of isotope-labeled internal standards, and then applied for the measurements via LC-ESI-MS/MS (LCMS-8060NX). Pellets obtained from centrifugation were dissolved in PBS containing 0.1% SDS for the determination of protein concentrations using the BCA assay. LC-ESI-MS/MS conditions and the synthesis of isotope-labeled internal standards were consistent with previously reported methods.[7] The results are shown in Figure 5 in the manuscript.

GAPDH Persulfidation Studies (with free -SH and NEM-blocked proteins): 216 μL of PBS (50 mM, pH 7.4) was added to 3.9 mg of GAPDH (from rabbit muscle, #G2267, Sigma-Aldrich) to obtain a concentration of 500 μM (MW: 36 kDa). DTT (10 mM in DMSO, 11.35 μL) was added for a final equal concentration with the GAPDH. The solution was incubated in the dark at rt for 30 min. Zeba 7K MWCO spin columns were prepared and used according to manufacturer's instructions with 3 PBS washes. Excess reductant was removed by column purification with the Zeba 7K MWCO columns. The resulting reduced protein concentration was determined by the absorbance at 280 nm (ε = 30.4 mM^{-1} cm^{-1}) using the Nanodrop One Microvolume UV-Vis Spectrophotometer (ThermoFisher Scientific). For controls, reduced GAPDH was treated with NEM (1:5 equiv.) and incubated at rt in the dark for 30 min before excess NEM was removed by the Zeba 7K MWCO columns. Next, the reduced protein was diluted to a final concentration of 100 μM in a final volume of 100 μL and treated with TTS (10 μL of 10 mM in DMSO for a final concentration of 1 mM), Na_2S (10 μL of 10 mM in MilliQ H_2O for a final concentration of 1 mM), both TTS and Na_2S, or Na_2S_2 (10 μL of 10 mM in MilliQ H_2O for a final concentration of 1 mM). *For example, if the reduced protein (361 μM) was only to be treated with TTS, 62.3 μL of PBS (50 mM, pH 7.4) would first be added to an Eppendorf tube, followed by 27.7 μL of protein, and followed by 10 μL of TTS.* The NEM-

treated proteins were treated with both TTS and Na_2S or Na_2S_2 (10 µL of 10 mM in MilliQ H_2O for a final concentration of 1 mM). TTS + Na_2S and Na_2S_2 samples were also prepared at the concentrations above without any protein, where PBS (50 mM, pH 7.4) was used in place of the proteins (volume-wise). Samples were incubated at rt for 30 min in the dark before being purified by Zeba 7K MWCO columns (three times to ensure proper removal of excess treatment molecules) and quantified by absorbance. For samples that did not contain protein (and couldn't be quantified by Nanodrop), volumes used followed those of their respective samples containing protein. All samples were then diluted in PBS (50 mM, pH 7.4) to a final protein concentration of 10 uM and incubated with SSP4 (5 µL of 100 µM in DMSO for a final concentration of 5 µM SSP4) in individual wells of a black clear flat bottom 96-well plate. The final volume was 100 µL. *For example, if the persulfidated protein (99.5 µM) was to be examined, 84.95 µL of PBS (50 mM, pH 7.4) would first be added to a well, followed by 10.05 µL of protein, and then followed by 5 µL of SSP4.* Incubation occurred at rt in the dark for 30 min before being measured on the SpectraMax iD3 microplate reader (ex: 485 nm; em: 525 nm). The results are shown in Supplementary Figure 9-A.

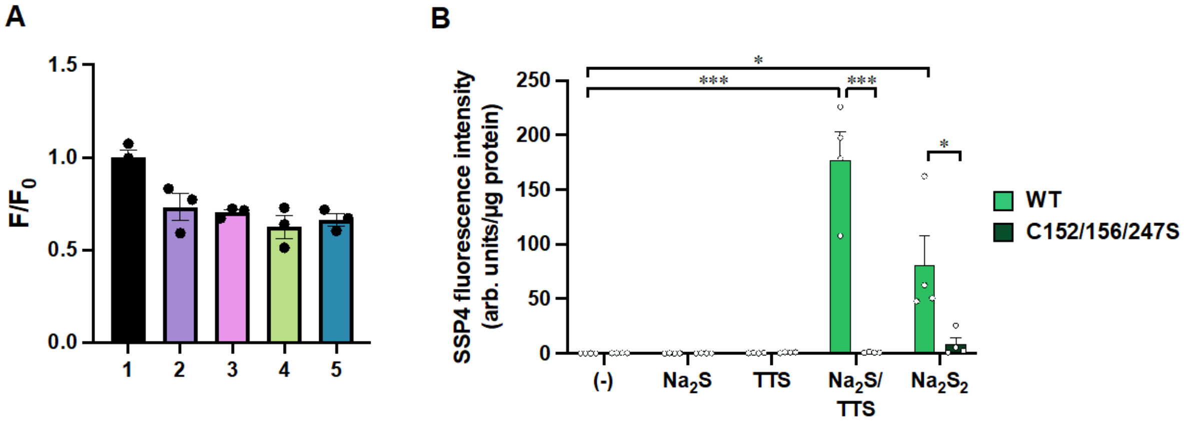

Supplementary Figure 9. A) Fluorescence responses of SSP4 (5 µM) for GAPDH (10 µM, after Zeba column desalting and dilution) when the reduced protein (100 µM) was treated with: (1) control (no treatment); NEM followed by (2) TTS (1 mM) + Na_2S (1 mM) or (3) Na_2S_2 (1 mM); or small molecules were added to Zeba columns without protein (4) TTS (1 mM) + Na_2S (1 mM); (5) Na_2S_2 (1 mM). Results are expressed as mean ± SEM (n = 3 distinct samples). B) SSP4 fluorescence for the detection of persulfidated GAPDH. WT GAPDH and C152/156/247S mutant (10 µM) were treated without or with 500 µM Na_2S, TTS, TTS + Na_2S,

or Na_2S_2, followed by desalting using a G-25 column and measurement of SSP4 fluorescence. Data are mean values ± SEM (n = 4 samples). *P = 0.0254, 0.0415. ***P = 0.00041, 0.00042. multiple t-test (two sided). Source data are provided as a Source Data file.

GAPDH Persulfidation Studies (with WT and mutant human GAPDH proteins): Preparation of recombinant human GAPDH: Human cDNA corresponding to GAPDH was obtained via PCR using a specific primer set (refer to Supplementary Table 6). The resulting amplified DNA fragment was then inserted to the NdeI and BamHI sites of the pET30a(+) vector. Using the pET30a(+)-hGAPDH WT plasmid as the template, a site-specific triple mutant of GAPDH, denoted as C152/156/247S, was constructed by using the QuikChange Site-Directed Mutagenesis kit (Stratagene Co., CA) following the manufacturer's protocol. PCR primers used for the mutagenesis process are provided in Supplementary Table 6.

The recombinant WT and Cys mutant of GAPDH were purified using the method we previously reported with some modifications.[7] Briefly, *E. coli* BL21 (DE3) transformed with pET30a(+)-hGAPDH WT or pET30a(+)-hGAPDH C152/156/247S were grown at 37 °C until reaching OD_{600} of 0.6-0.7. Subsequently, induction was carried out by the addition of 1 mM isopropyl-β-D-thiogalactopyranoside (Sigma-Aldrich) and followed by incubation for 3 h at 37 °C. The hGAPDH proteins were purified using Ni-NTA agarose (Qiagen, Hilden, Germany) according to the manufacturer's instructions. The purified proteins were desalted using a NAP-5 column with 20 mM Tris-HCl buffer (pH 7.5) containing 1 mM TCEP and 150 mM NaCl, and then stored at -80 °C. Protein concentration was determined using the Protein Assay CBB Solution (Nacalai Tesque), and the purity of the proteins was confirmed via SDS-PAGE.

SSP4 assay: Purified recombinant GAPDH protein was applied to a PD SpinTrap G-25 column pre-equilibrated with phosphate-buffered saline (PBS) at pH 7.4 to eliminate reductants. The proteins were quantified (10 μM) using the Bradford protein assay, and subsequently, incubated in PBS including 500 μM Na_2S, 500 μM TTS, 500 μM TTS + 500 μM Na_2S or 500 μM Na_2S_2 in the dark for 30 min at room temperature. The samples were desalted using a PD SpinTrap G-25 column to remove excess treatment molecules. The treated samples were then subjected to a reaction with 5 μM SSP4 in PBS (pH 7.5) containing 250 μM cetyltrimethylammonium

bromide (CTAB) in the dark for 30 min at room temperature. Subsequently, the fluorescence intensities of the resulting solutions were measured using a microplate reader (SH-9000, Corona Electric Co., Ltd.) at an excitation wavelength of 488 nm and an emission wavelength of 521 nm. The results are shown in Supplementary Figure 9-B.

Supplementary Table 6. Primer sequences for the construction of WT GAPDH and Cys mutant GAPDH expression vectors.

Primer name	Sequence (5′- 3′)
pET30a(+)-hGAPDH WT Fw	CAT ATG GGG AAG GTG AAG GTC GGA GT
pET30a(+)-hGAPDH WT Rv	GGA TCC TTA CTC CTT GGA GGC CAT GT
hGAPDH C152S Fw	TCAGCAATGCCTCCAGCACCACCAACTGC
hGAPDH C152S Rv	GCAGTTGGTGGTGCTGGAGGCATTGCTGA
hGAPDH C156S Fw	CCTGCACCACCAACAGCTTAGCACCCCTG
hGAPDH C156S Rv	CAGGGGTGCTAAGCTGTTGGTGGTGCAGG
hGAPDH C247S Fw	GTGGTGGACCTGACCAGCCGTCTAGAAAAAC
hGAPDH C247S Rv	GTTTTTCTAGACGGCTGGTCAGGTCCACCAC

Recognition sequences of the restriction enzyme are underlined with one heavy line.
Mutant bases are underlined with double lines.

LC-MS/MS of Persulfidated GAPDH and Data Analysis Procedure: The undiluted protein sample treated with TTS and Na_2S (in "GAPDH Persulfidation Studies (with free -SH and NEM-blocked proteins)") was then treated with ~20 equivalents of HPE-IAM to prepare the sample for Tandem MS analysis. HPE-IAM was prepared in DMSO to a stock concentration of 10 mM. 10 uL of HPE-IAM (10 mM) was added to the protein sample followed by 50 mM PBS (pH 7.4) to increase the volume to a final volume of 100 μL. After incubating in the dark at rt for 1 h, the sample was purified by the Zeba 7K MWCO column. The sample was then quantified by Nanodrop. The sample was then taken up in S-Trap lysis buffer (5% SDS, 50 mM TEAB, pH 7.5) and acidified to a pH <1. Seven times the sample volume of binding/wash buffer (10% 50 mM TEAB, pH 7.5, 90% MeOH) was then added to the sample and mixed by pipetting. The supernatant was transferred to the S-Trap column with a 2.0 mL receiver tube

for waste flowthrough. The S-Trap column was then centrifuged at 4,000xg for 30 seconds to trap the proteins. After, 200 µL of binding/wash buffer was added, and the sample was centrifuged at 4,000xg for 30 seconds. This was repeated four times and flowthrough was discarded. The S-Trap micro column was then transferred to a clean 1.6 mL tube for digestion. 20 µL of digestion buffer containing 1:10 ratio mass/mass trypsin was added and incubated overnight at 37°C in a covered belly dancer water bath. After digestion, 40 µL of 50 mM TEAB was added to the S-Trap and then centrifuged (4,000 rcf, 1 min), then 40 µL of 0.2% FA was added to the S-Trap and centrifuged (4,000 rcf, 1 min), and then 40 µL of 50% ACN was added to the S-Trap and centrifuged (4,000 rcf, 1 min). The elution was then dried down and taken up in 40 µL MilliQ H_2O and the BCA Assay was performed. The protein concentration was adjusted to 0.05 µg/µL in MilliQ H_2O. Samples were then submitted for MS, and MSGF+ (DMS) peptide identification was performed.

LC-MS/MS was performed with a nanoAcquity ultra performance liquid chromatography (UPLC) system coupled to an in-house prepared 25 cm x 75 µm i.d. 1.7 µm Waters BEH C18 column with a flow rate of 200 nL/min using a 90 min. gradient from H_2O to acetonitrile (ACN) with constant 0.1% formic acid. Mass spectrometry was performed using electrospray ionization on a Q-Exactive Plus Orbitrap Mass Spectrometer (Thermo Scientific, San Jose, CA). Full MS spectra were recorded at a resolution of 70 k over a range of m/z 300-1800 with an automated gain control (AGC) value of 3 x 10^6. MS/MS was performed in data dependent mode (DDA) by selecting the top 12 parent ions for fragmentation and subsequent analysis at 17.5 k resolution with AGC target of 1 x 10^5. Tandem MS was performed using high-energy collision dissociation (HCD) with a normalized collision energy setting of 30. Precursor ion activation was performed using an isolation window of 1.5 m/z with an intensity threshold of 1 x 10^5.

LC-MS/MS raw data files were converted to mzML format using MSConvert (ProteoWizard) before performing peptide identification using the MSGF+ algorithm (v2023.01.12).[8,9] MS/MS spectra were searched against the full rabbit protein sequence database (UniProt, downloaded March 7, 2021). Key parameters include a precursor mass tolerance of 20 ppm, static modifications: none, dynamic modifications: methionine oxidation (+15.9949 Da), ß-(4-

hydroxyphenyl)ethyl iodoacetamide (HPE-IAM) alkylated cysteine (+177.19988), HPE-IAM alkylated cysteine persulfide (+209.05105), and HPE-IAM alkylated trisulfide (+241.02311). Trypsin digestion rules were applied to a partially tryptic target-decoy database search. Data processing was performed by parsing the mzid file, performing protein inference, and filtering peptide IDs in R (4.1.3) using the MSnID package (1.25.2).[10] Filtering parameters for absolute ppm error and $-\log_{10}$(MSGF SpecEValue) were optimized with final values of <1.9 and >9.6, respectively. False discovery rate (FDR) was restricted to 1% at the protein level.

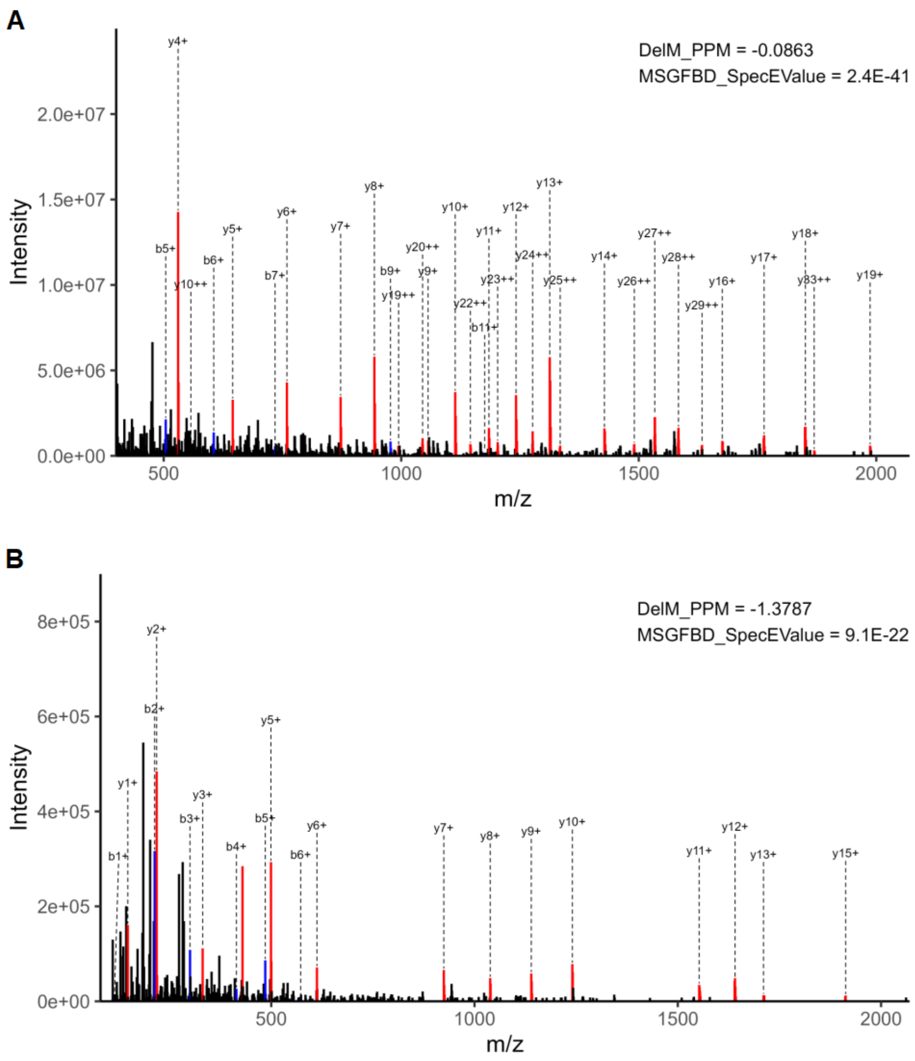

Supplementary Figure 10. Tandem mass spectra (MS2): (a) Spectrum of a GAPDH peptide GILGYTEDQVVSC(282)@DFNSDTHSSTFDAGAGIALNDHFVK persulfidated at Cys282 with the TTS + Na_2S system. Fragment ions containing persulfidation include y26++, y27++, y28++, and y29++. (b) Spectrum of a GAPDH peptide

IVSNASC(150)@TTNC(154)@LAPLAK persulfidated at both Cys150 and Cys154 with the TTS + Na_2S system. Fragment ions containing persulfidation include y11+, y12+, y13+, and y15+. The symbol @ indicates persulfidation.

Cell viability and cytotoxicity assay:

HeLa cells were cultured in DMEM/F12 (1:1) (Gibco, Invitrogen, #11330-032) medium supplemented with 10% fetal bovine serum (FBS) at 37°C, 5% CO_2 overnight. Cells were then seeded at a density of 11,000 cells/well in the inner wells of a 96-well black/clear flat-bottomed plate (ThermoFisher Scientific, #165305) at 37°C, 5% CO_2 overnight. Media was then aspirated from wells, and cells were washed twice with 1X PBS. TTS and TT were first prepared at 10, 25, 50, and 100 mM concentrations in DMSO and then diluted in 10% FBS-containing media to 10, 25, 50, and 100 µM (0.1% DMSO). Compounds were then added to cells (100 µL/well) and incubated for 24 hrs at 37°C, 5% CO_2. Afterwards, WST-8 from the CCK-8 assay was added to cells (10 µL) following the manufacturer's instructions and incubated for 2 hrs at 37°C, 5% CO_2. The absorbance at 450 nm was measured with the Molecular Devices SpectraMax iD3 Multi-Mode Microplate Reader. The optical density (OD) of the wells (3 wells per condition) was used to calculate the relative cell viability (%) according to the following formula:

Cell Viability (%) = (ODtreatment group - OD_{blank} / ODcontrol group - OD_{blank}) * 100

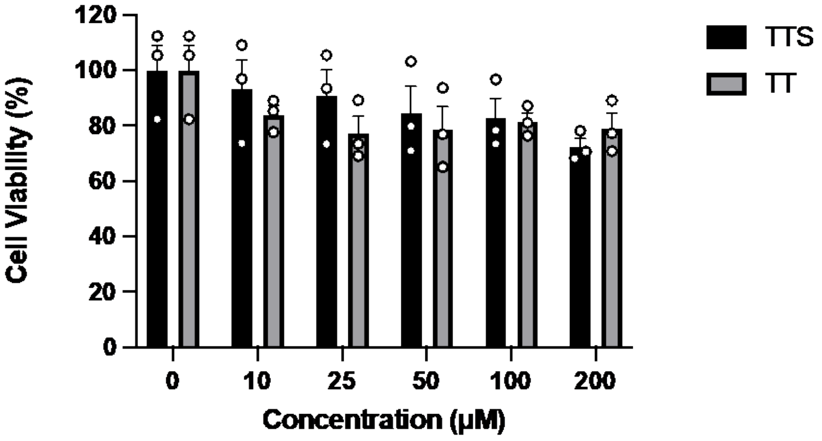

Supplementary Figure 11. Cell viability of TTS and TT in HeLa cells with the CCK-8 assay. Cells were treated with different concentrations of TTS and TT (0-100 µM) and incubated for 24 hrs at 37°C, 5% CO_2. After the initial incubation period, WST-8 was added, and cells were incubated for another 2 hrs at 37°C, 5% CO2. Absorbance was measured at 450 nm. Results are expressed as mean ± SEM (n = 3 distinct wells containing cells). Source data are provided as a Source Data file.

UV-Vis absorbance study of the reaction between TTS and Papain: Papain was reduced with L-cysteine and purified following literature procedures.[11] After purification, papain was quantified by Nanodrop (246.18 µM) using the ε_{mM} = 57.6 (280 nm). The Thermo Scientific Evolution 300 UV-Vis Spectrophotometer was set to scan every 1 minute. TTS (10 mM stock in DMSO) was mixed with reduced papain (246.18 µM) at 1:1 and 1:4 equivalents in PBS (50 mM, pH 7.4) with a pipette and immediately placed in the UV-Vis Spectrophotometer for scanning. The results are shown in Supplementary Figure 12 below. We did not observe significant changes to the TTS's UV-Vis spectra. These suggest that TTS was stable in the presence of papain.

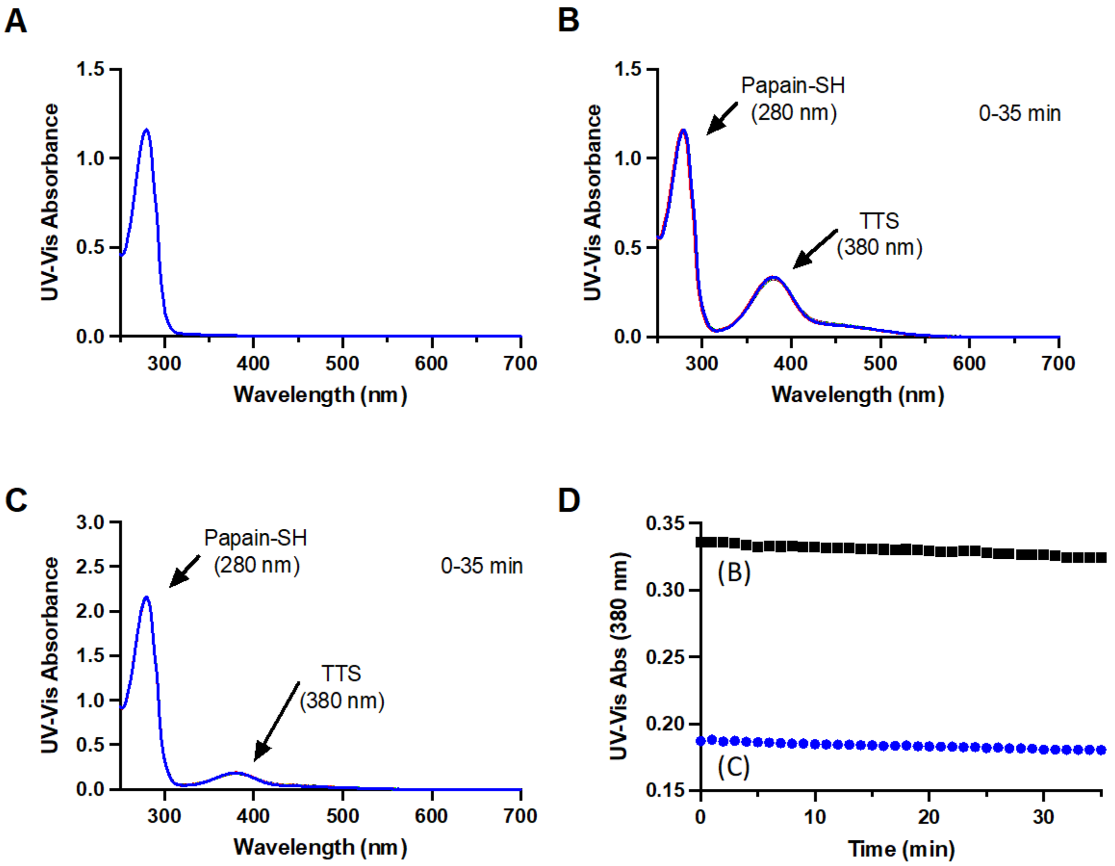

Supplementary Figure 12. UV-Vis absorbance spectra of: A**)** reduced papain (20 µM); B**)** TTS (20 µM) and reduced papain (20 µM) over 35 min (1 min/scan); and C**)** TTS (10 µM) and reduced papain (40 µM) over 35 min (1 min/scan). D**)** Time-dependent UV-Vis absorbance changes at 380 nm of reactions B**)** and C**)** over 35 min. Source data are provided as a Source Data file.

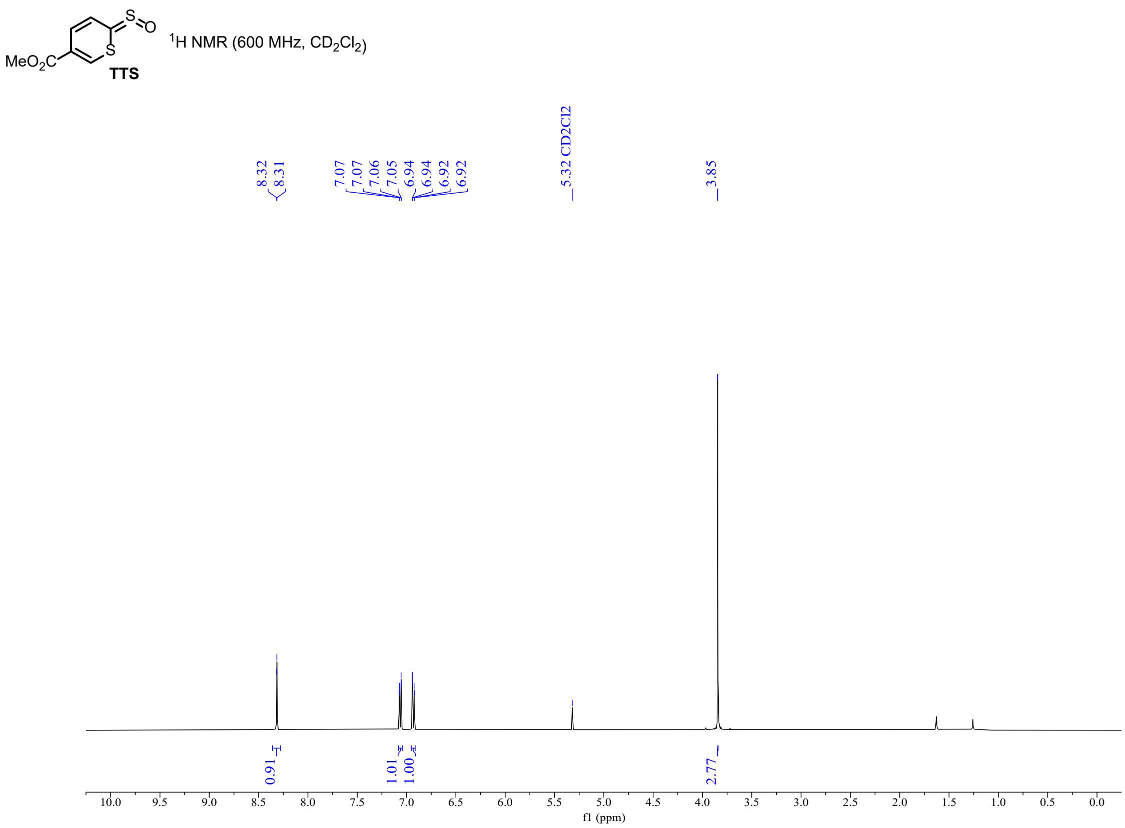

Supplementary Figure 13. ^{1}H-NMR spectrum of TTS.

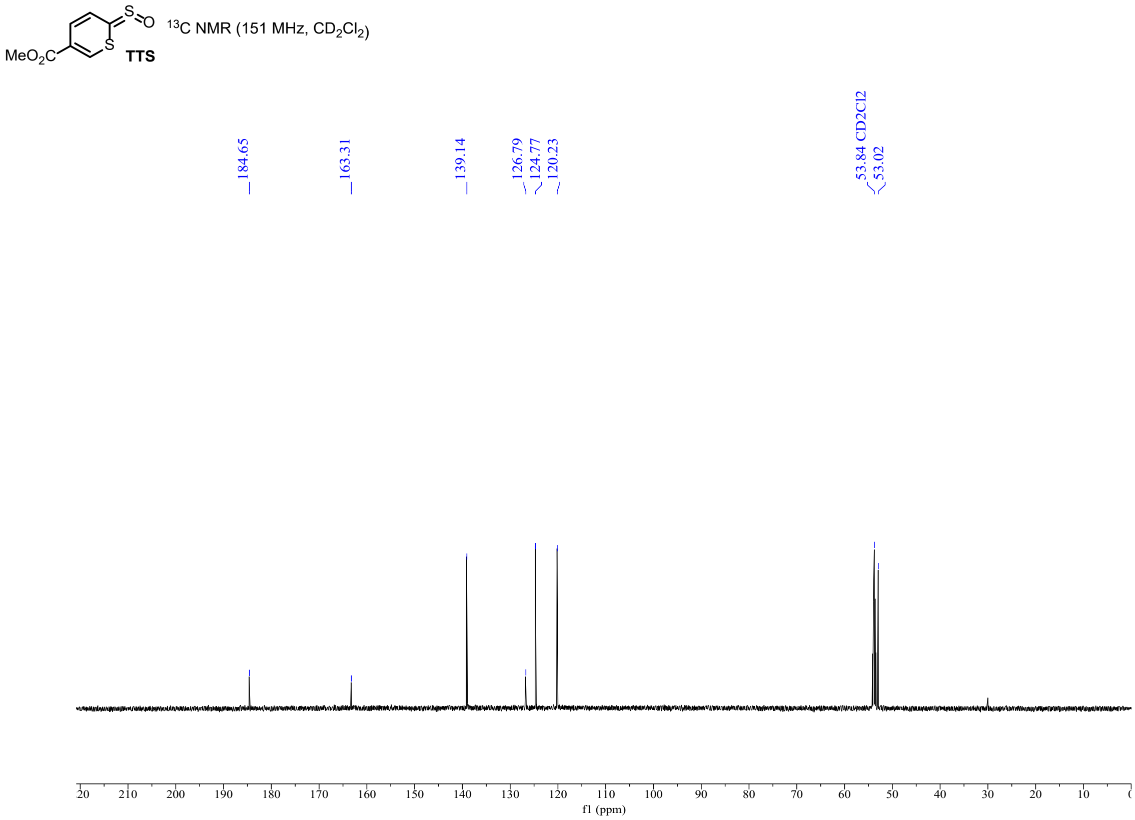

Supplementary Figure 14. ^{13}C-NMR spectrum of TTS.

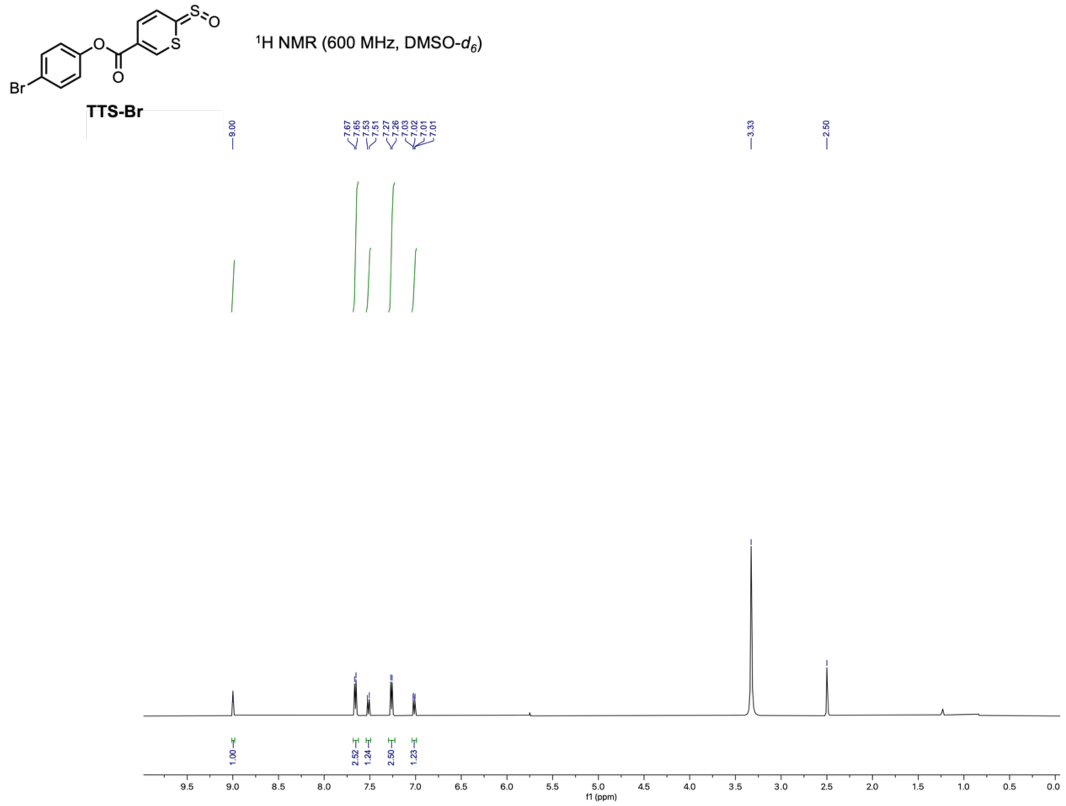

Supplementary Figure 15. ^{1}H-NMR spectrum of TTS-Br.

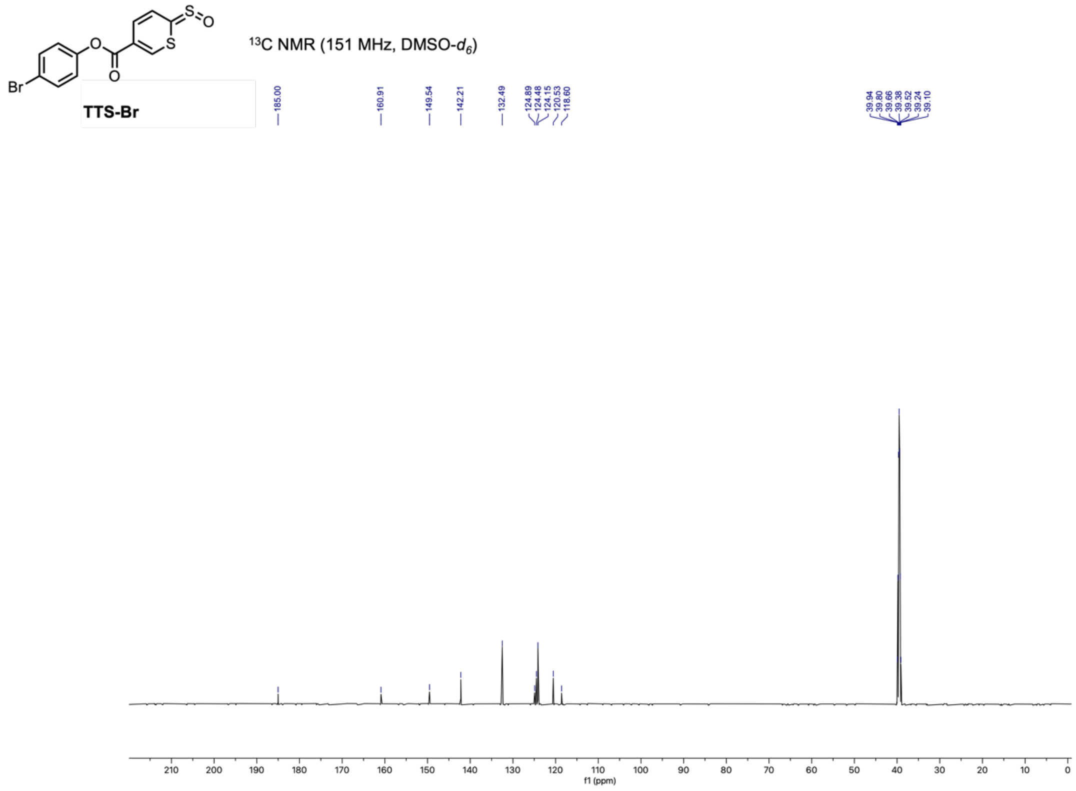

Supplementary Figure 16. ^{13}C-NMR spectrum of TTS-Br.

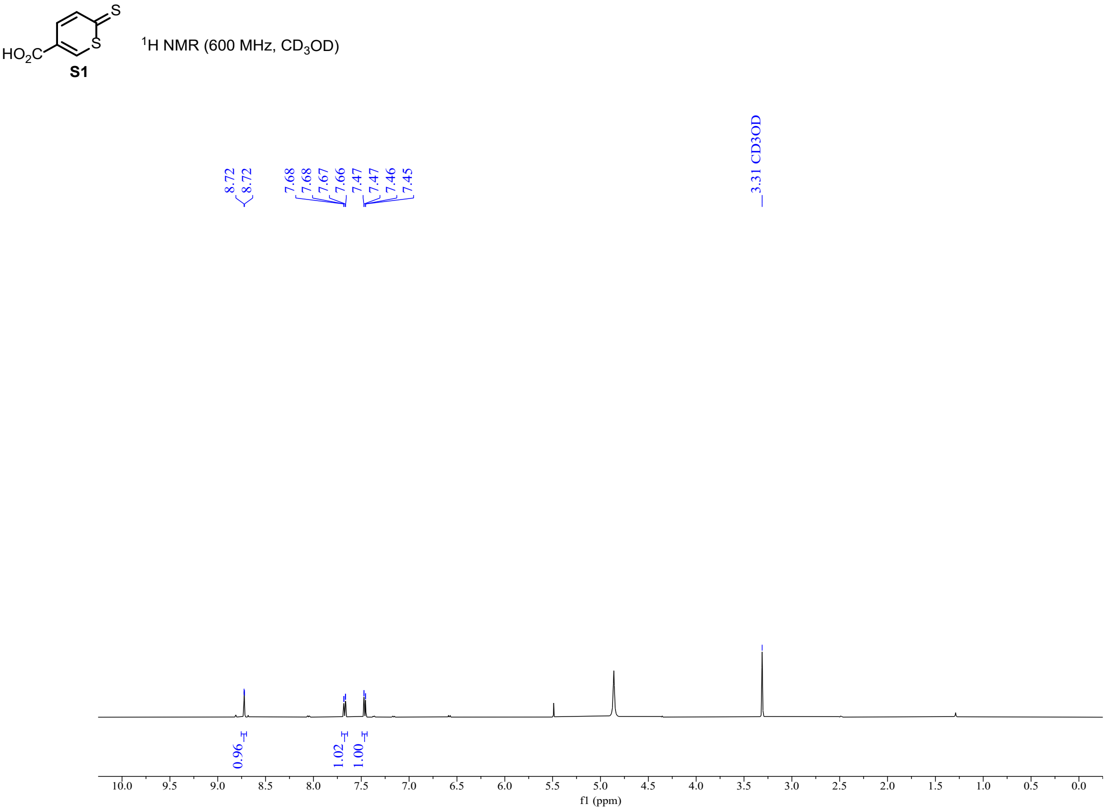

Supplementary Figure 17. ^{1}H-NMR spectrum of compound S1.

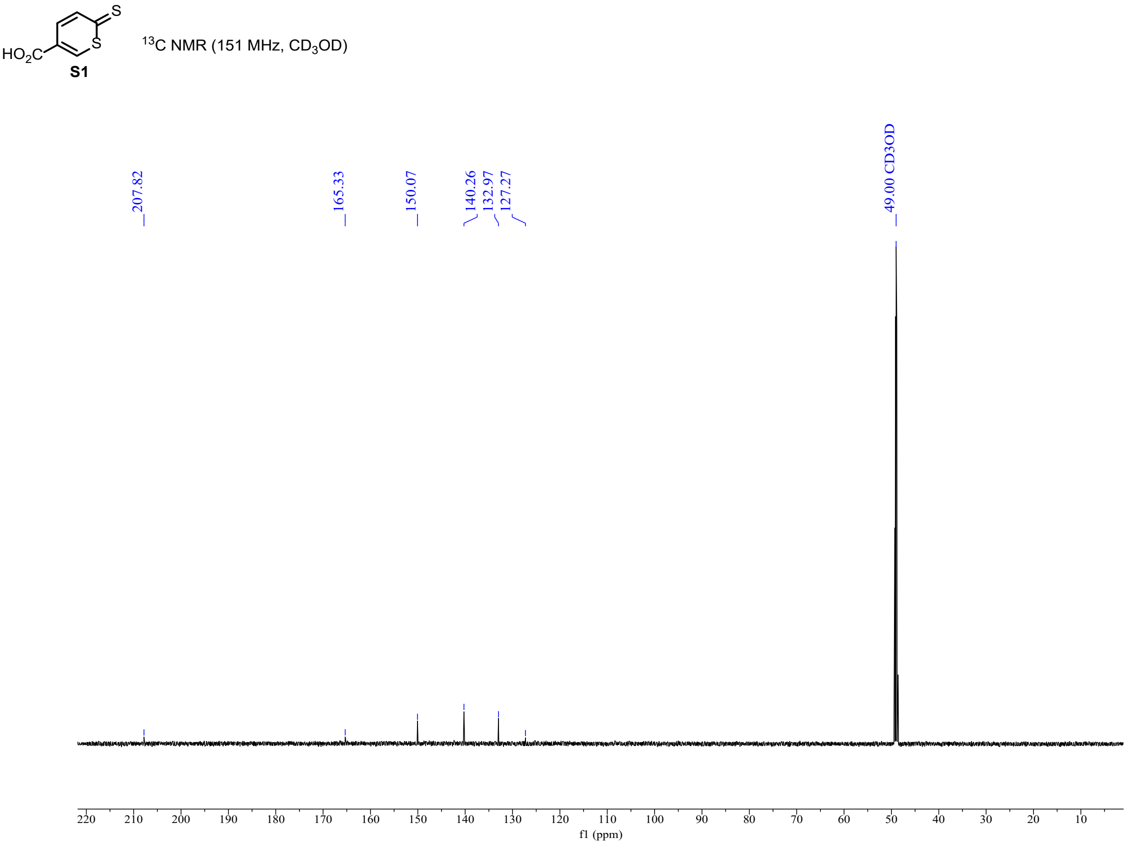

Supplementary Figure 18. ^{13}C-NMR spectrum of compound S1.

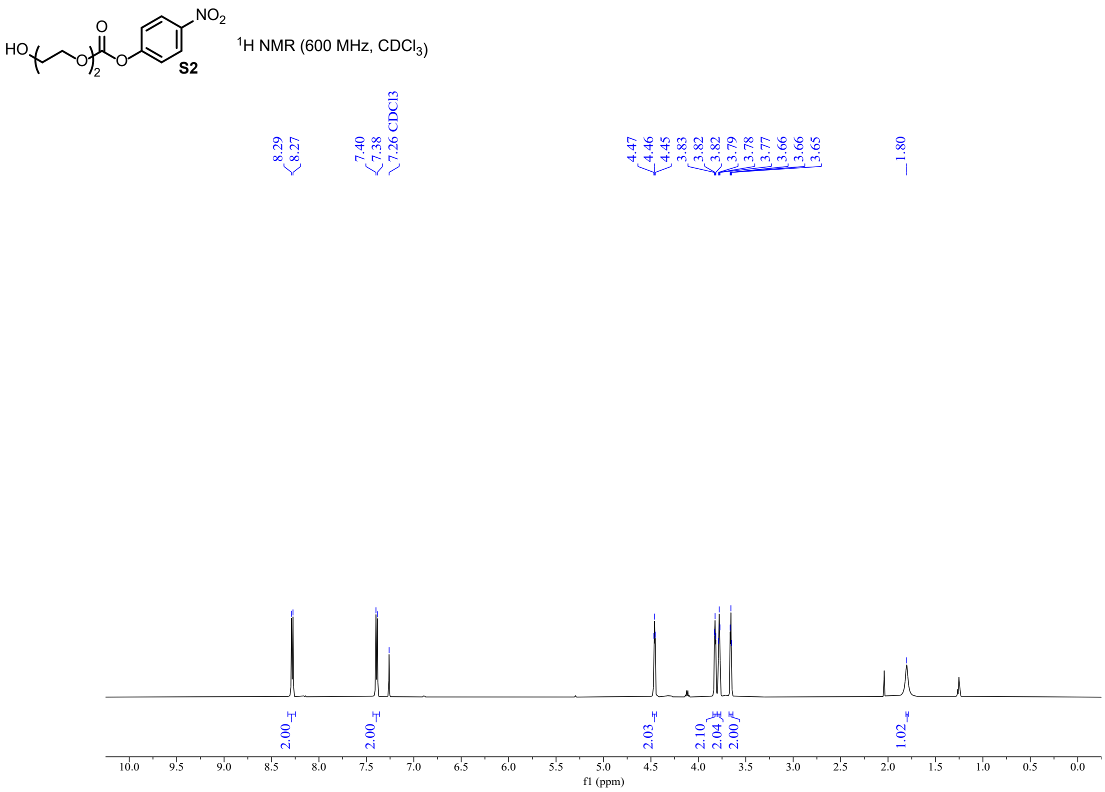

Supplementary Figure 19. ^{1}H-NMR spectrum of compound S2.

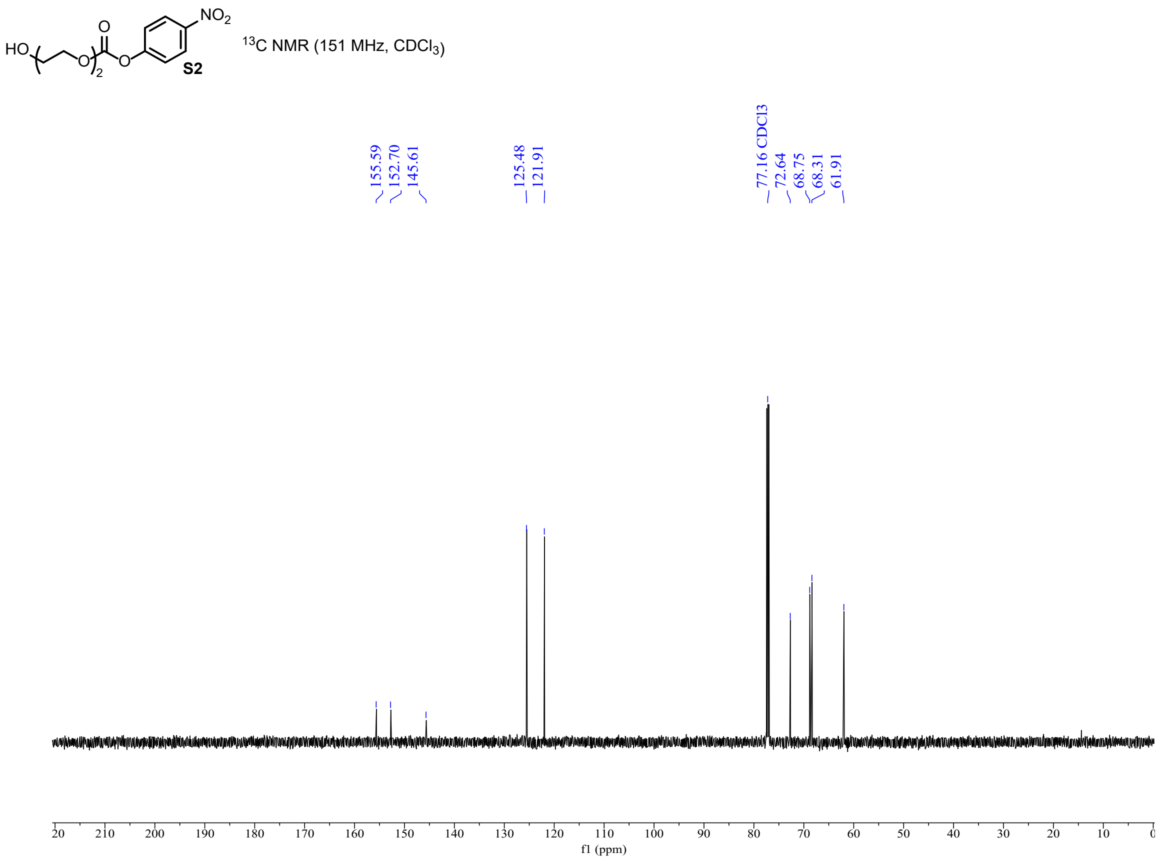

Supplementary Figure 20. ^{13}C-NMR spectrum of compound S2.

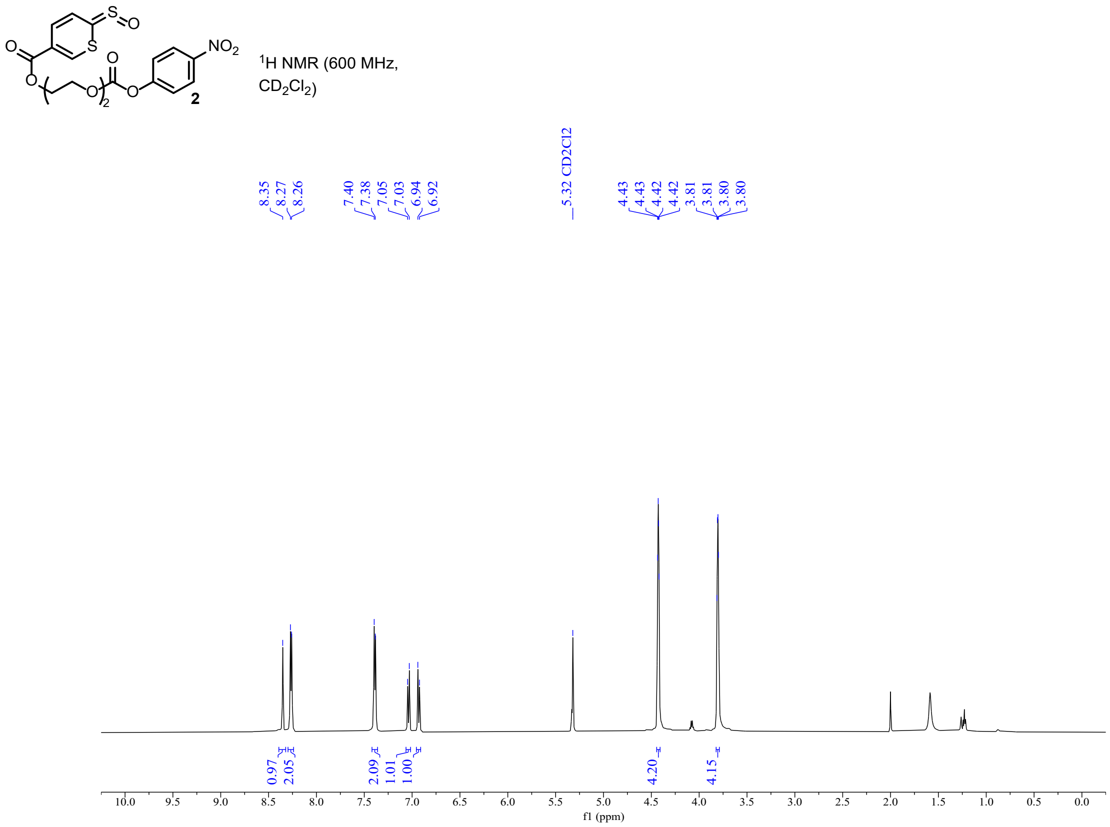

Supplementary Figure 21. ^{1}H-NMR spectrum of compound **2**.

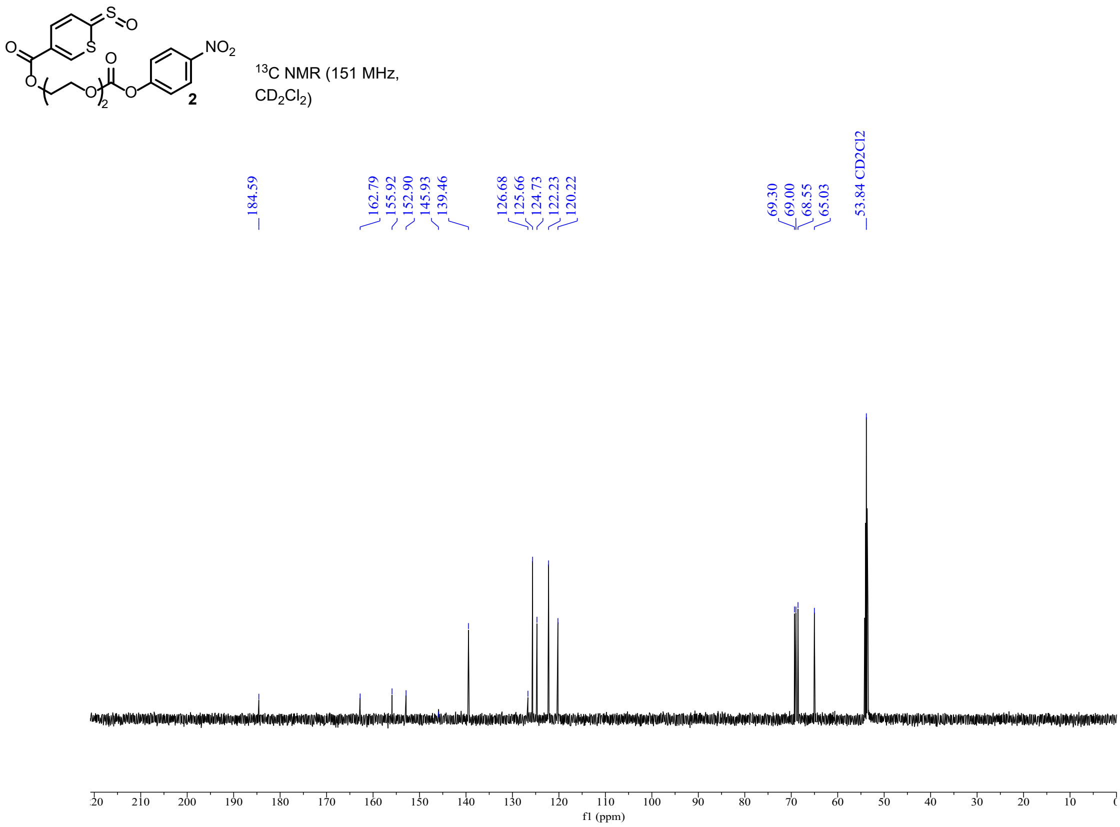

Supplementary Figure 22. ^{13}C-NMR spectrum of compound **2**.

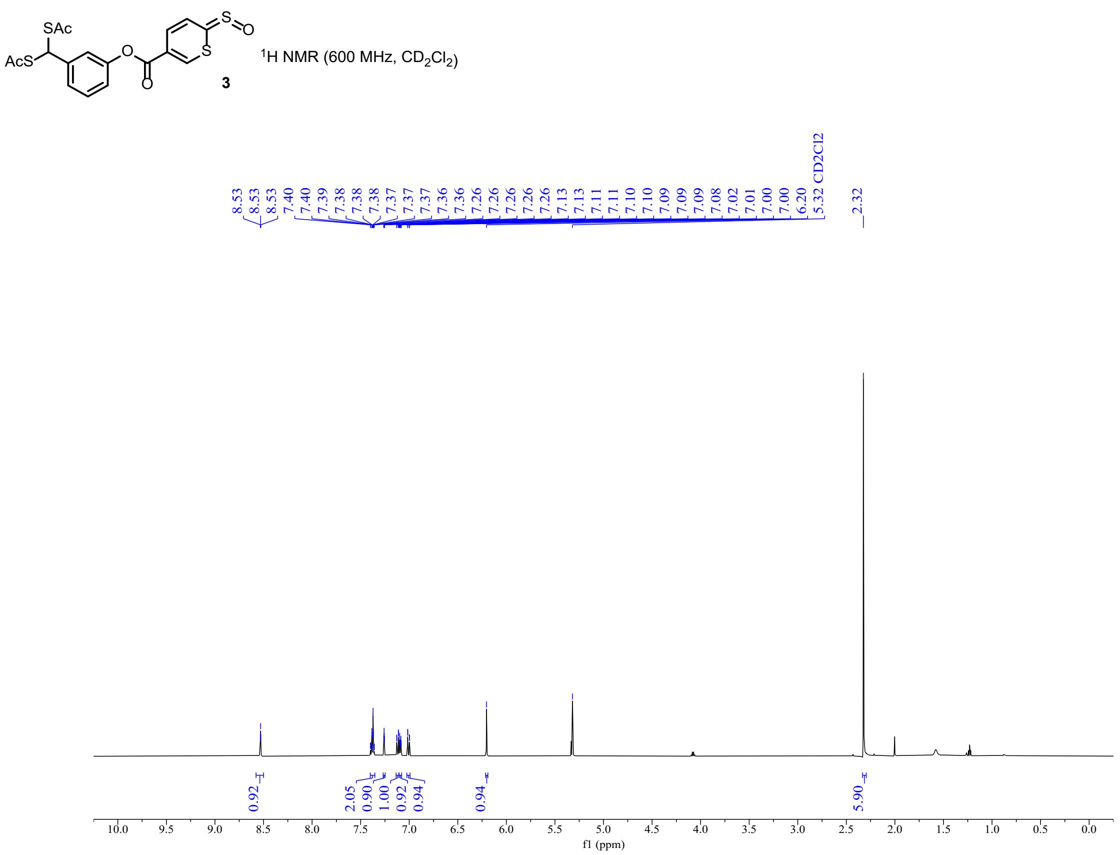

Supplementary Figure 23. ^{1}H-NMR spectrum of compound **3**.

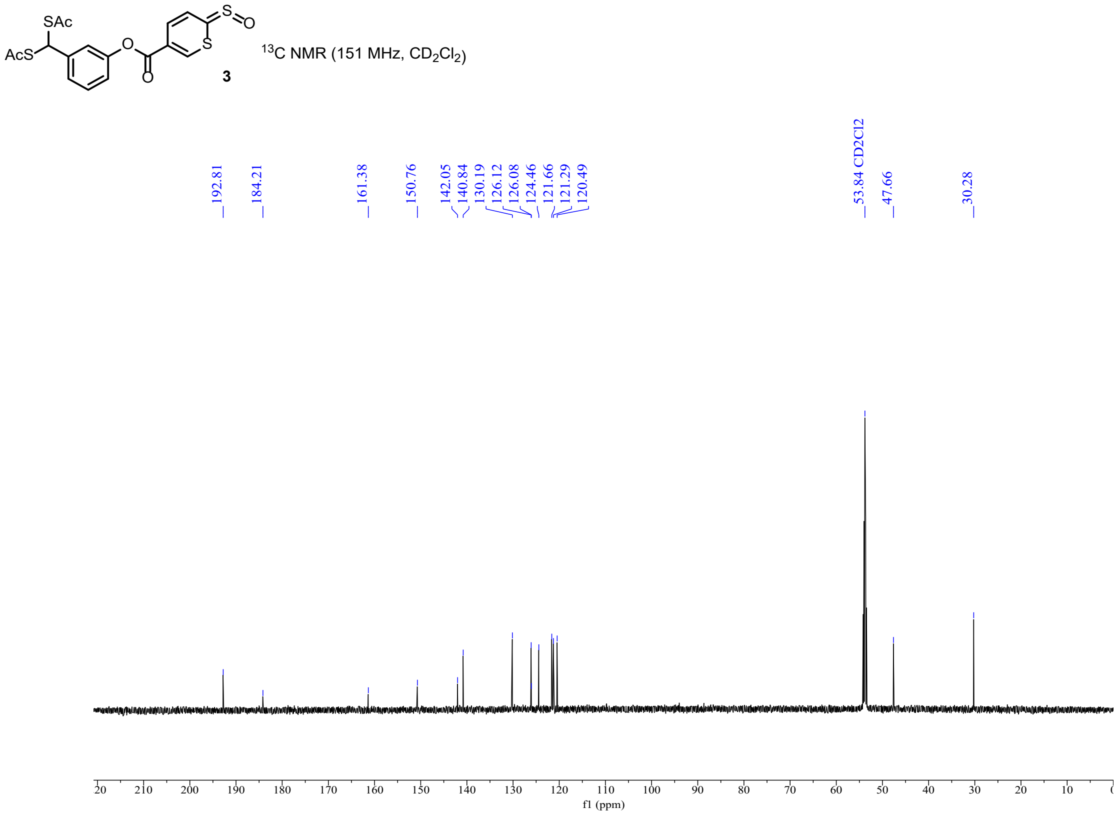

Supplementary Figure 24. ^{13}C-NMR spectrum of compound **3**.

Supplementary References

1. Defoina, A.; Augelmann, G.; Fritzb, H.; Geffroy, G.; Schmidlina, C.; Streitha, J., *Helv. Chim. Acta,* **1985**, *68*, 1998.

2. Zhao, Y.; Kang, J.; Park, C. M.; Bagdon, P. E.; Xian, M. *Org. Lett*. **2014**, *16*, 4536.

3. SAINT, version 8.37a; Bruker AXS Inc.: Madison, WI, 2012.

4. Sheldrick, G. *Acta Crystallogr., Sect. A: Found. Adv.* **2015**, *71*, 3−8.

5. SADABS, version 2014/5; Bruker AXS Inc.: Madison, WI, **2001**.

6. Sheldrick, G. *Acta Crystallogr., Sect. C: Struct. Chem*. **2015**, *71*, 3−8.

7. Akaike, T. et al. *Nat. Commun.* **2017**, *8*, 1177.

8. Kim, S.; Pevzner, P. A. *Nat. Commun.* **2014,** *5*, 5277.

9. Chambers, M. C.; Maclean, B.; Burke, R.; Amodei, D.; Ruderman, D. L.; Neumann, S.; Gatto, L.; Fischer, B.; Pratt, B.; Egertson, J.; Hoff, K.; Kessner, D.; Tasman, N.; Shulman, N.; Frewen, B.; Baker, T. A.; Brusniak, M-Y.; Paulse, C.; Creasy, D.; Flashner, L.; Kani, K.; Moulding, C.; Seymour, S. L.; Nuwaysir, L. M.; Lefebvre, B.; Kuhlmann, F.; Roark, J.; Rainer, P.; Detlev, S.; Hemenway, T.; Huhmer, A.; Langridge, J.; Connolly, B.; Chadick, T.; Holly, K.; Eckels, J.; Deutsch, E. W.; Moritz, R. L.; Katz, J. E.; Agus, D. B.; MacCoss, M.; Tabb, D. L.; Mallick, P. *Nat. Biotechnol.* **2012**, *30*, 918-920.

10. Petyuk, V.; Gatto, L. *MSnID: Utilities for Exploration and Assessment of Confidence of LC-MSn Proteomics Identifications*. **2023**, R package version 1.25.2.

11. Shieh, M.; Ni, X.; Xu, S.; Lindahl, S. P.; Yang, M.; Matsunaga, T.; Flaumenhaft, R. C.; Akaike, T.; Xian, M. *Redox Biol*. **2022**, *56*, 102433.
