# Supplementary material for: 2H-Thiopyran-2-thione sulfine, a compound for converting H2S to HSOH/H2S2 and increasing intracellular sulfane sulfur levels
Source: Nat Commun. 2024 Mar 19;15:2453. doi: 10.1038/s41467-024-46652-7 (PMC10951338; doi:10.1038/s41467-024-46652-7)
Supplement: Supplementary file 2 — Reporting Summary [file 41467_2024_46652_MOESM2_ESM.pdf]

nature portfolio

Corresponding author(s): Ming Xian, Takaaki Akaike

Last updated by author(s): Feb 8, 2024

Reporting Summary

Nature Portfolio wishes to improve the reproducibility of the work that we publish. This form provides structure for consistency and transparency in reporting. For further information on Nature Portfolio policies, see our Editorial Policies and the Editorial Policy Checklist.

Statistics

For all statistical analyses, confirm that the following items are present in the figure legend, table legend, main text, or Methods section.

n/a	Confirmed	
☐	☒	The exact sample size (*n*) for each experimental group/condition, given as a discrete number and unit of measurement
☐	☒	A statement on whether measurements were taken from distinct samples or whether the same sample was measured repeatedly
☐	☒	The statistical test(s) used AND whether they are one- or two-sided<br>*Only common tests should be described solely by name; describe more complex techniques in the Methods section.*
☒	☐	A description of all covariates tested
☐	☒	A description of any assumptions or corrections, such as tests of normality and adjustment for multiple comparisons
☐	☒	A full description of the statistical parameters including central tendency (e.g. means) or other basic estimates (e.g. regression coefficient) AND variation (e.g. standard deviation) or associated estimates of uncertainty (e.g. confidence intervals)
☐	☒	For null hypothesis testing, the test statistic (e.g. *F*, *t*, *r*) with confidence intervals, effect sizes, degrees of freedom and *P* value noted<br>*Give P values as exact values whenever suitable.*
☒	☐	For Bayesian analysis, information on the choice of priors and Markov chain Monte Carlo settings
☒	☐	For hierarchical and complex designs, identification of the appropriate level for tests and full reporting of outcomes
☐	☒	Estimates of effect sizes (e.g. Cohen's *d*, Pearson's *r*), indicating how they were calculated

Our web collection on statistics for biologists contains articles on many of the points above.

Software and code

Policy information about availability of computer code

Data collection	Gaussian 09, SpectraMax iD3 Multi-Mode Microplate Reader (Molecular Devices)
Data analysis	GraphPad Prism (v. 9 and v. 10.0.3)(GraphPadSoftware, La Jolla, CA), MestReNova (v. 14), imaging data were analyzed using Fiji (National Institutes of Health) software or ImageJ (National Institutes of Health)

For manuscripts utilizing custom algorithms or software that are central to the research but not yet described in published literature, software must be made available to editors and reviewers. We strongly encourage code deposition in a community repository (e.g. GitHub). See the Nature Portfolio guidelines for submitting code & software for further information.

Data

Policy information about availability of data

All manuscripts must include a data availability statement. This statement should provide the following information, where applicable:

- Accession codes, unique identifiers, or web links for publicly available datasets
- A description of any restrictions on data availability
- For clinical datasets or third party data, please ensure that the statement adheres to our policy

Data generated and analyzed in this study are included in this article and in its Supplementary Information files. Source data are provided with this paper. Accession code: CCDC 2259484 contains the supplementary crystallographic data for this paper. These data can be obtained free of charge via www.ccdc.cam.ac.uk/data_request/cif, or by emailing data_request@ccdc.cam.ac.uk, or by contacting The Cambridge Crystallographic Data Centre, 12 Union Road, Cambridge CB2 1EZ, UK; fax: +44 1223 336033. The protein mass spectrometry data have been deposited to the MassIVE repository with the dataset accession: MSV000094034. The

data will also be available through the ProteomeXchange with the accession number: PXD049256.

Research involving human participants, their data, or biological material

Policy information about studies with human participants or human data. See also policy information about sex, gender (identity/presentation), and sexual orientation and race, ethnicity and racism.

Reporting on sex and gender	n/a
Reporting on race, ethnicity, or other socially relevant groupings	n/a
Population characteristics	n/a
Recruitment	n/a
Ethics oversight	n/a

Note that full information on the approval of the study protocol must also be provided in the manuscript.

Field-specific reporting

Please select the one below that is the best fit for your research. If you are not sure, read the appropriate sections before making your selection.

☒ Life sciences ☐ Behavioural & social sciences ☐ Ecological, evolutionary & environmental sciences

For a reference copy of the document with all sections, see nature.com/documents/nr-reporting-summary-flat.pdf

Life sciences study design

All studies must disclose on these points even when the disclosure is negative.

Sample size	No sample size calculation was performed. Sample sizes were chosen based on previous experience, previous published works, and on what is common practice in the field.. The number of independently performed experiments is indicated in the figure legends and methods.
Data exclusions	No data were excluded from analysis.
Replication	All experiments were independently repeated as described in the legends and methods and were reliably reproduced. The number of independently performed experiments is indicated in the figure legends and methods.
Randomization	No experimental groups were used in this study because they were in vitro studies, so randomization was not relevant.
Blinding	Group allocation and blinding were not relevant to this study.

Reporting for specific materials, systems and methods

We require information from authors about some types of materials, experimental systems and methods used in many studies. Here, indicate whether each material, system or method listed is relevant to your study. If you are not sure if a list item applies to your research, read the appropriate section before selecting a response.

Materials & experimental systems

n/a	Involved in the study	
☒	☐	Antibodies
☐	☒	Eukaryotic cell lines
☒	☐	Palaeontology and archaeology
☐	☒	Animals and other organisms
☒	☐	Clinical data
☒	☐	Dual use research of concern
☒	☐	Plants

Methods

n/a	Involved in the study	
☒	☐	ChIP-seq
☒	☐	Flow cytometry
☒	☐	MRI-based neuroimaging

Eukaryotic cell lines

Policy information about cell lines and Sex and Gender in Research

Cell line source(s)	HeLa cells were obtained from ATCC. Neonatal rat cardiomyocytes (NRCMs) were isolated from Sprague-Dawley rat pups (including male and female) on postnatal day 2. Immortalized mouse embryonic fibroblasts (MEFs) were obtained from mouse embryos. The SQR Knockdown MEFs were produced according to the shRNA-mediated knockdown method reported recently(doi: 10.1038/s41467-023-40182-4.)
Authentication	Cell lines were not authenticated.
Mycoplasma contamination	Cell lines tested negative for mycoplasma contamination.
Commonly misidentified lines (See ICLAC register)	No commonly misidentified cell lines were used in this study.

Animals and other research organisms

Policy information about studies involving animals; ARRIVE guidelines recommended for reporting animal research, and Sex and Gender in Research

Laboratory animals	Sprague-Dawley rat pups (male and female) on postnatal day 2 were purchased from Japan SLC, Inc.
Wild animals	This study did not involve wild animals.
Reporting on sex	Neonatal rat cardiomyocytes were isolated from both male and female rat pups.
Field-collected samples	This study did not involve samples collected from the field.
Ethics oversight	Protocol (code: 23A015) using rats were reviewed and approved by the ethics committees at the National Institutes of Natural Sciences.

Note that full information on the approval of the study protocol must also be provided in the manuscript.

Plants

Seed stocks	n/a
Novel plant genotypes	n/a
Authentication	n/a
