# Supplementary material for: 2H-Thiopyran-2-thione sulfine, a compound for converting H2S to HSOH/H2S2 and increasing intracellular sulfane sulfur levels
Source: Nat Commun. 2024 Mar 19;15:2453. doi: 10.1038/s41467-024-46652-7 (PMC10951338; doi:10.1038/s41467-024-46652-7)
Supplement: Supplementary file 4 — Description of Additional Supplementary files [file 41467_2024_46652_MOESM4_ESM.pdf]

Description of Additional Supplementary files

File name: Supplementary Data 1
Description: The Cartesian coordinates of all computed structures
