# Supplementary material for: 2H-Thiopyran-2-thione sulfine, a compound for converting H2S to HSOH/H2S2 and increasing intracellular sulfane sulfur levels
Source: Nat Commun. 2024 Mar 19;15:2453. doi: 10.1038/s41467-024-46652-7 (PMC10951338; doi:10.1038/s41467-024-46652-7)
Supplement: Supplementary file 5 — Supplementary Data 1 [file 41467_2024_46652_MOESM5_ESM.pdf]

Cartesian coordinates of all computed structures

TT

C -1.12541200 0.25541700 0.00007700
C -0.53723600 -0.95789400 -0.00001700
C 1.80358700 0.36391000 -0.00002600
C 0.97543600 1.52189200 0.00005600
C -0.37391200 1.47496400 0.00010700
H -1.11030100 -1.87531300 -0.00002300
H 1.47650600 2.48214700 0.00009600
H -0.92921000 2.40196100 0.00018400
S 3.46321600 0.49239600 -0.00002600
C -2.61067800 0.36809400 0.00016100
O -3.18877200 1.43201800 0.00016600
O -3.22922500 -0.80362500 0.00002000
C -4.66497900 -0.75779600 0.00000000
H -4.99006500 -1.79202200 -0.00014000
H -5.01799000 -0.24525300 0.89185500
H -5.01795300 -0.24502500 -0.89173900
S 1.15542000 -1.23953300 -0.00004700
O 4.02924900 -0.92637000 -0.00033800

HS-

S 0.00000000 0.00000000 0.07894100
H 0.00000000 0.00000000 -1.26305500

TS1

C 1.36968400 0.03651200 -0.39588900
C 0.80247900 -1.06561100 0.13737100
C -1.55482000 0.12699100 -0.37738500
C -0.73251700 1.04884800 -1.13532700
C 0.60787800 1.01867200 -1.13062400
H 1.39620700 -1.84000700 0.60393300
H -1.25662400 1.82188300 -1.68547800
H 1.15810400 1.76413100 -1.68681800
S -3.21119300 0.08638600 -0.77467400
C 2.83865000 0.22312800 -0.32594000
O 3.42630400 1.10924500 -0.91123600
O 3.45573400 -0.66959100 0.44296200
C 4.88062600 -0.53940800 0.54307500
H 5.20172300 -1.34012400 1.20021900
H 5.33616800 -0.64841100 -0.43866000
H 5.13782200 0.42753100 0.96935800
S -0.87357900 -1.42482300 0.10336100
O -3.83043000 -1.10344300 0.01459400
S -1.42113600 1.29924700 1.83698100
H -2.56360800 0.65753900 2.12452300

INT1

C 1.40078000 0.18803500 -0.30512100
C 0.85591500 -1.04489200 -0.31301400
C -1.54196400 0.25178800 0.01756500
C -0.72378600 1.39644300 -0.49349400

C 0.60727900 1.37552300 -0.57794800
H 1.46707800 -1.93596600 -0.25562800
H -1.27290200 2.29882100 -0.74268300
H 1.13798800 2.26507800 -0.88796700
S -3.20894200 0.43449500 -0.66801400
C 2.85967300 0.36038200 -0.15474700
O 3.42402600 1.42998600 -0.27657100
O 3.51113100 -0.76629800 0.13330200
C 4.93299000 -0.65321100 0.27554300
H 5.28456300 -1.65197500 0.51032700
H 5.37752100 -0.30415600 -0.65390400
H 5.17427200 0.03321800 1.08410700
S -0.83091900 -1.35259800 -0.51460900
O -3.99531800 -0.86948500 -0.21983900
S -1.52212600 0.18574200 1.87751000
H -2.04076200 1.41473500 2.03972700

INT2

C 1.47277000 0.18870600 -0.24624700
C 0.94627700 -1.05549700 -0.29781300
C -1.51611900 0.21323900 0.02758800
C -0.67685500 1.37698400 -0.40002700
C 0.65966500 1.37111900 -0.45622300
H 1.57173700 -1.93769100 -0.26348500
H -1.21727000 2.30211300 -0.56537900
H 1.17921700 2.29651300 -0.66318400
S -3.10566200 0.33150800 -0.90926200
C 2.92529500 0.37765100 -0.09262100
O 3.48121800 1.45555900 -0.19352300
O 3.59449100 -0.74668200 0.17583800
C 5.01385600 -0.61615600 0.31947200
H 5.37950500 -1.61273300 0.54234700
H 5.45506100 -0.25045500 -0.60527400
H 5.24740100 0.06348100 1.13613000
S -0.72164100 -1.37374100 -0.52657000
O -4.07743300 -0.57297300 0.08841400
S -1.84904400 0.24710400 1.79384600
H -3.72962600 -0.36266200 1.00001300

TS2

C -1.36972800 -0.42467000 -0.12405000
C -0.83192100 -0.24952600 1.10325800
C 1.57776100 -0.78858300 -0.07484600
C 0.73092900 -1.09790900 -1.20028800
C -0.60678300 -0.93892100 -1.22811300
H -1.42769000 0.06232800 1.95048800
H 1.24677800 -1.45625300 -2.08174400
H -1.14462000 -1.18277800 -2.13350000
S 1.72334400 1.82281000 -0.75694300
C -2.81073600 -0.15353100 -0.35266000
O -3.38095400 -0.41044200 -1.39226600
O -3.41939000 0.40466400 0.68913800
C -4.81403700 0.69749400 0.52482700

H -5.12605600 1.16589300 1.45175100
H -4.95614100 1.37822500 -0.31145900
H -5.37158300 -0.22117700 0.35655200
S 0.80667600 -0.56265400 1.48494700
O 3.05588300 1.94632700 0.32236600
S 3.22125400 -1.17226100 -0.00289600
H 3.46170100 1.05691700 0.32352600

TTS

C 0.79399900 0.21724500 0.00001300
C 0.27364700 -1.03176400 0.00001400
C -2.19810600 0.17617300 0.00009000
C -1.39108500 1.35255100 0.00002400
C -0.03504400 1.37617400 0.00000800
H 0.89454800 -1.91813500 0.00000400
H -1.93249400 2.28908700 -0.00001400
H 0.46194600 2.33665800 -0.00001800
C 2.26794500 0.42415600 -0.00000500
O 2.78036800 1.52145200 0.00000200
O 2.95674600 -0.70816500 0.00001600
C 4.38702600 -0.57711100 0.00001800
H 4.77211400 -1.59058800 0.00002600
H 4.70998900 -0.04493700 -0.89183000
H 4.70998500 -0.04492400 0.89185900
S -1.39777200 -1.37046500 0.00002000
S -3.85868400 0.17309000 -0.00009200

HOS-

H -0.88860700 1.37305200 0.00000000
S 0.03702500 -0.64405500 0.00000000
O 0.03702500 1.11647800 0.00000000

HSS-

S 0.03996600 -1.01042200 0.00000000
H -1.27891000 -1.26925500 0.00000000
S 0.03996600 1.08975000 0.00000000

H_2S

S 0.00000200 0.10275400 -0.00000600
H -0.97179900 -0.82201600 0.00001100
H 0.97176800 -0.82204600 0.00009100

H_2O

H -0.76095700 -0.47114700 0.00000500
H 0.76095500 -0.47114800 0.00005800
O 0.00000000 0.11778700 -0.00000800

TTS

Compound Table

Label	Tgt Score	Mass Error (ppm)	Tgt Formula	Obs. RT	Ref. Mass	Obs. Mass
Cpd 1: C7 H6 O3 S2; 0.513	65.9	2.7	C7 H6 O3 S2	0.513	201.97584	201.97638

Obs. m/z	Obs. RT	Obs. Mass	Tgt Formula	Tgt Mass	Tgt Mass Error (ppm)	Find Cpds Algorith
202.98364	0.513	201.97638	C7 H6 O3 S2	201.97584	2.7	Find by Formula

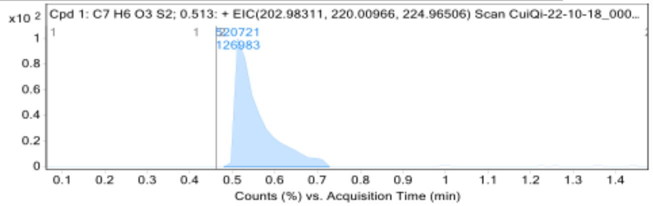

MS Zoomed Spectrum

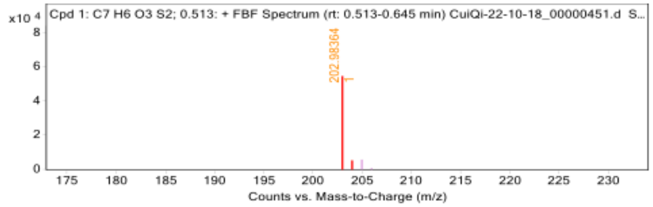

MS Zoomed Spectrum

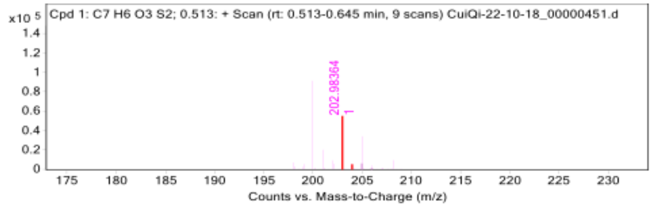

MS Spectrum Peak List

Obs. m/z	Charge	Abund	Formula	Ion/Isotope	Tgt Mass Error (ppm)
202.98364	1	53758.71	C7H6O3S2	(M+H)+	
203.98658	1	5641.71	C7H6O3S2	(M+H)+	
202.98364	1	53758.71	C7H6O3S2	(M+H)+	2.58
203.98658	1	5641.71	C7H6O3S2	(M+H)+	3.68

--- End Of Report ---

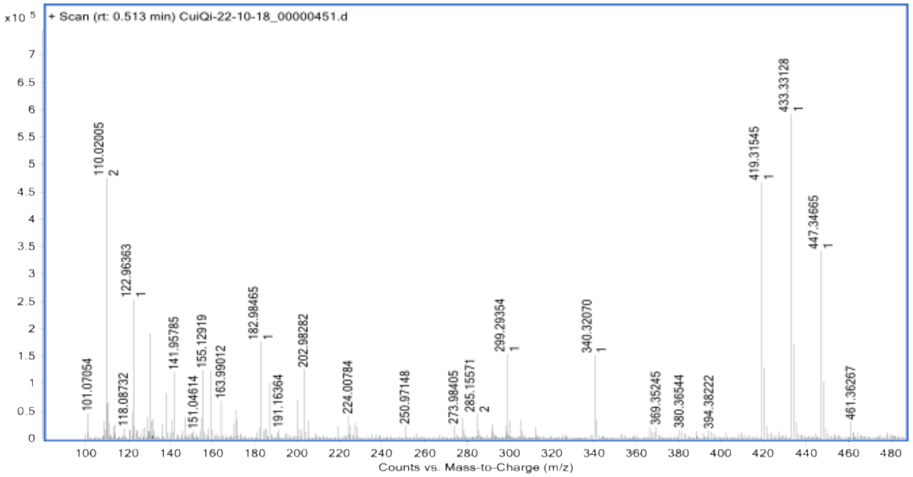

TTS-Br

Compound Table

Label	Tgt Score	Mass Error (ppm)	Tgt Formula	Obs. RT	Ref. Mass	Obs. Mass
Cpd 1: C12 H7 Br O3 S2; 0.394	74.72	8.7	C12 H7 Br O3 S2	0.394	341.902	341.90497

Obs. m/z	Obs. RT	Obs. Mass	Tgt Formula	Tgt Mass	Tgt Mass Error (ppm)	Find Cpds Algorith
342.91291	0.394	341.90497	C12 H7 Br O3 S2	341.902	8.7	Find by Formula

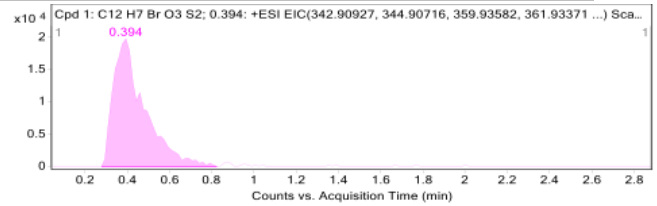

MS Zoomed Spectrum

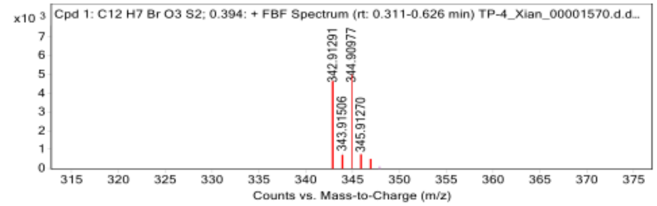

MS Zoomed Spectrum

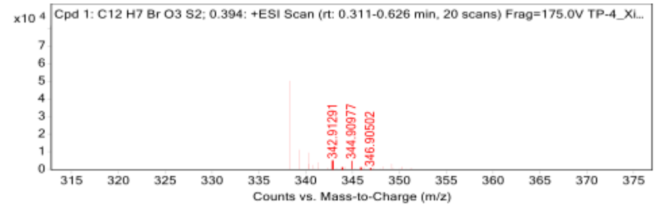

MS Spectrum Peak List

Obs. m/z	Charge	Abund	Formula	Ion/Isotope	Tgt Mass Error (ppm)
342.91291	1	4840.75	C12H7BrO3S2	(M+H)+	
343.91506	1	820.17	C12H7BrO3S2	(M+H)+	
344.90977	1	4632.91	C12H7BrO3S2	(M+H)+	
345.9127	1	761.22	C12H7BrO3S2	(M+H)+	
346.90502	1	450.8	C12H7BrO3S2	(M+H)+	
342.91291	1	4840.75	C12H7BrO3S2	(M+H)+	10.61
343.91506	1	820.17	C12H7BrO3S2	(M+H)+	8.24
344.90977	1	4632.91	C12H7BrO3S2	(M+H)+	7.56
345.9127	1	761.22	C12H7BrO3S2	(M+H)+	7.55
346.90502	1	450.8	C12H7BrO3S2	(M+H)+	1.47

--- End Of Report ---

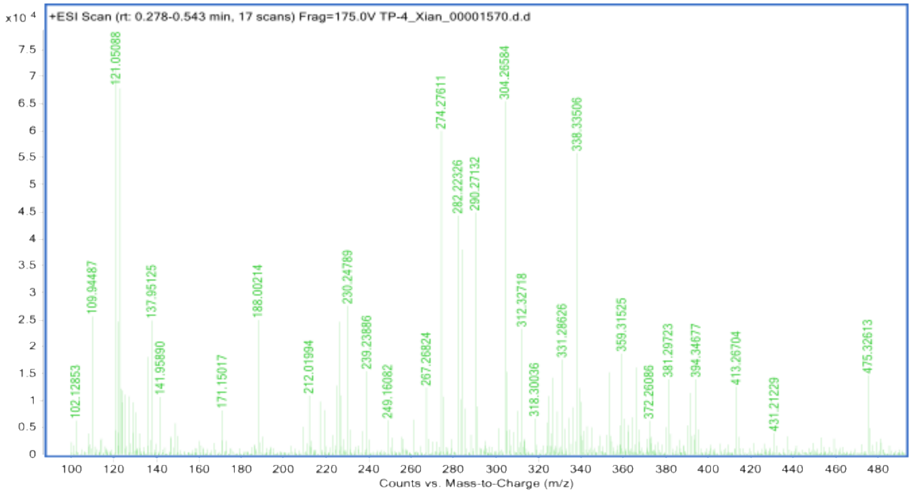

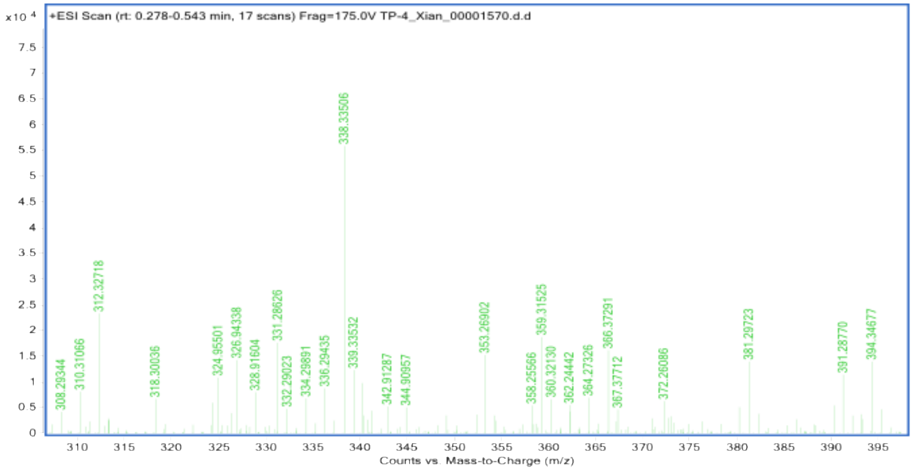

S1

Compound Table

Label	Tgt Score	Mass Error (ppm)	Tgt Formula	Obs. RT	Ref. Mass	Obs. Mass
Cpd 1: C6 H4 O2 S2; 0.594	82.04	-6.35	C6 H4 O2 S2	0.594	171.96527	171.96418

Obs. m/z	Obs. RT	Obs. Mass	Tgt Formula	Tgt Mass	Tgt Mass Error (ppm)	Find Cpds Algorith
172.97177	0.594	171.96418	C6 H4 O2 S2	171.96527	-6.35	Find by Formula

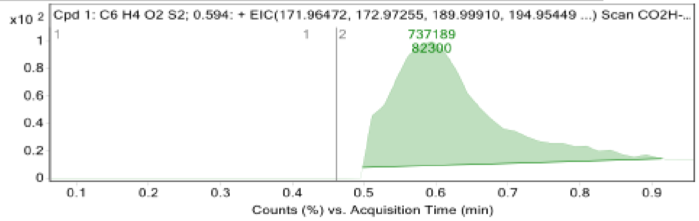

MS Zoomed Spectrum

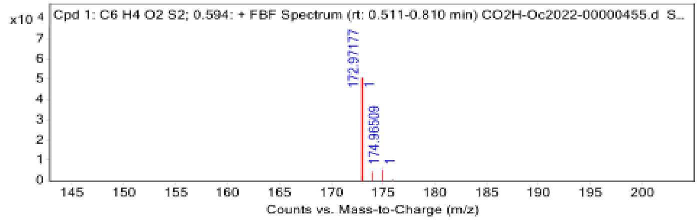

MS Zoomed Spectrum

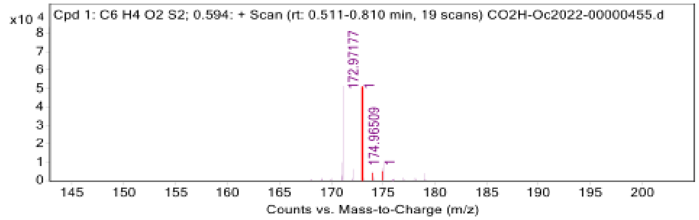

MS Spectrum Peak List

Obs. m/z	Charge	Abund	Formula	Ion/Isotope	Tgt Mass Error (ppm)
172.97177	1	48237.01	C6H4O2S2	(M+H)+	
173.97489	1	4693.43	C6H4O2S2	(M+H)+	
174.96509	1	6799.9	C6H4O2S2	(M+H)+	
172.97177	1	48237.01	C6H4O2S2	(M+H)+	-4.49
173.97489	1	4693.43	C6H4O2S2	(M+H)+	-1.59
174.96509	1	6799.9	C6H4O2S2	(M+H)+	-22.29

--- End Of Report ---

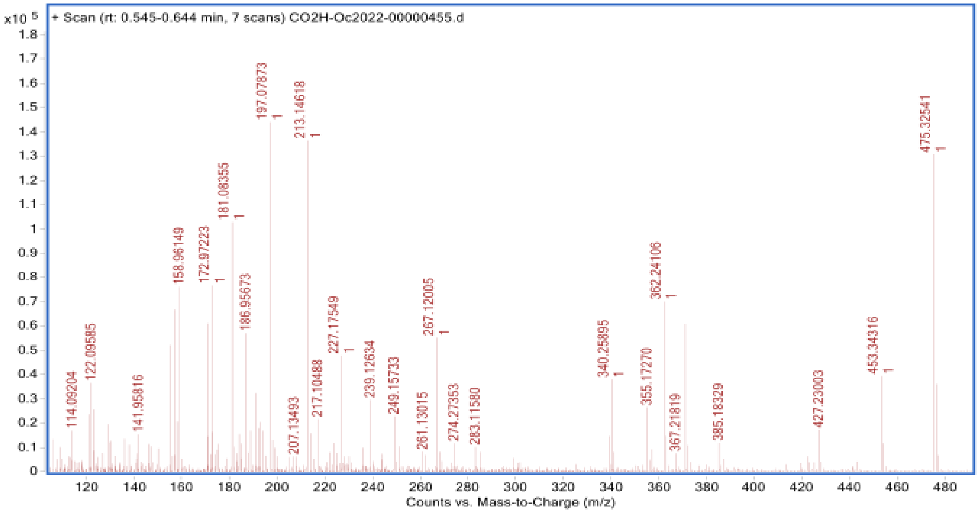

S2

Compound Table

Label	Tgt Score	Mass Error (ppm)	Tgt Formula	Obs. RT	Ref. Mass	Obs. Mass
Cpd 1: C11 H13 N O7; 0.495	99.88	0.41	C11 H13 N O7	0.495	271.0692	271.06931

Obs. m/z	Obs. RT	Obs. Mass	Tgt Formula	Tgt Mass	Tgt Mass Error (ppm)	Find Cpds Algorith
272.07664	0.495	271.06931	C11 H13 N O7	271.0692	0.41	Find by Formula

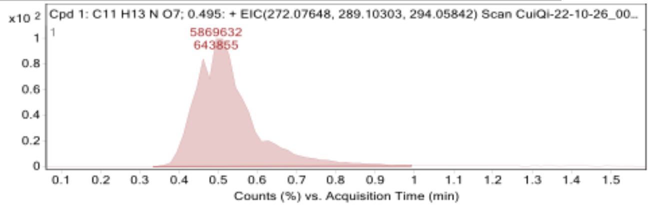

MS Zoomed Spectrum

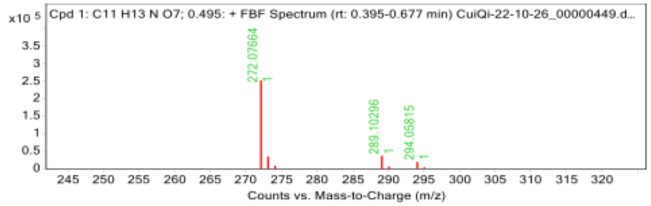

MS Zoomed Spectrum

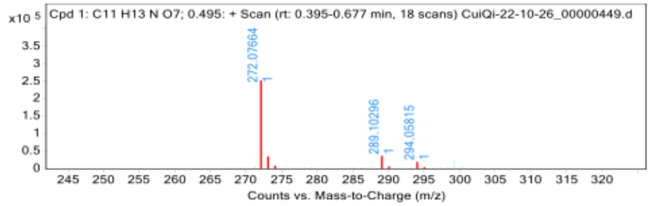

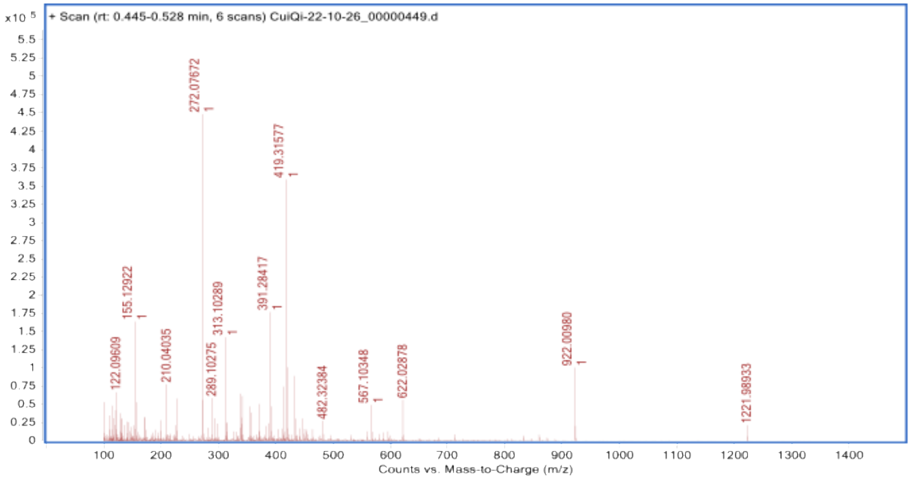

MS Spectrum Peak List

Obs. m/z	Charge	Abund	Formula	Ion/Isotope	Tgt Mass Error (ppm)
272.07664	1	251657.75	C11H13NO7	(M+H)+	
273.07985	1	31694.83	C11H13NO7	(M+H)+	
274.08204	1	5540.62	C11H13NO7	(M+H)+	
275.08621	1	555.23	C11H13NO7	(M+H)+	
289.10296	1	34847.55	C11H13NO7	(M+NH4)+	
290.10611	1	4973.66	C11H13NO7	(M+NH4)+	
291.10377	1	743.51	C11H13NO7	(M+NH4)+	
294.05815	1	18355.18	C11H13NO7	(M+Na)+	
295.06174	1	2463.42	C11H13NO7	(M+Na)+	
296.06107	1	396.62	C11H13NO7	(M+Na)+	
272.07664	1	251657.75	C11H13NO7	(M+H)+	0.6
273.07985	1	31694.83	C11H13NO7	(M+H)+	0.54
274.08204	1	5540.62	C11H13NO7	(M+H)+	2.07
275.08621	1	555.23	C11H13NO7	(M+H)+	7.22
289.10296	1	34847.55	C11H13NO7	(M+NH4)+	-0.22
290.10611	1	4973.66	C11H13NO7	(M+NH4)+	0.07
291.10377	1	743.51	C11H13NO7	(M+NH4)+	-14.29
294.05815	1	18355.18	C11H13NO7	(M+Na)+	-0.94
295.06174	1	2463.42	C11H13NO7	(M+Na)+	0.32
296.06107	1	396.62	C11H13NO7	(M+Na)+	-7.91

--- End Of Report ---

2

Compound Table

Label	Tgt Score	Mass Error (ppm)	Tgt Formula	Obs. RT	Ref. Mass	Obs. Mass
Cpd 1: C17 H15 N O9 S2; 0.519	85.68	2.67	C17 H15 N O9 S2	0.519	441.01882	441.02

Obs. m/z	Obs. RT	Obs. Mass	Tgt Formula	Tgt Mass	Tgt Mass Error (ppm)	Find Cpds Algorith
442.02623	0.519	441.02	C17 H15 N O9 S2	441.01882	2.67	Find by Formula

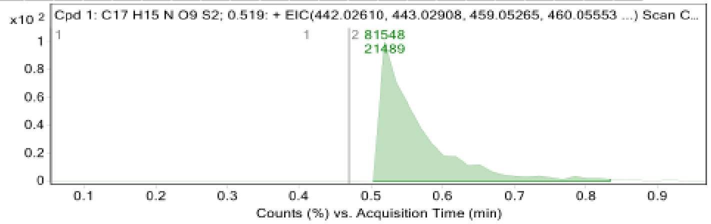

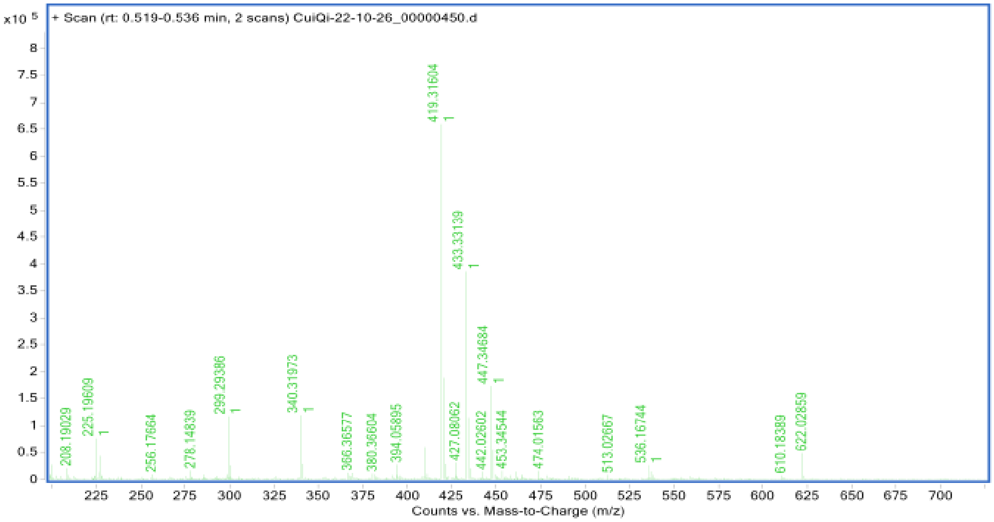

MS Zoomed Spectrum

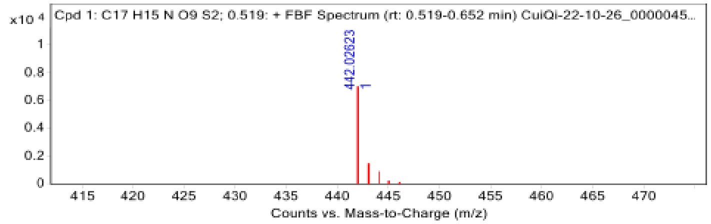

MS Zoomed Spectrum

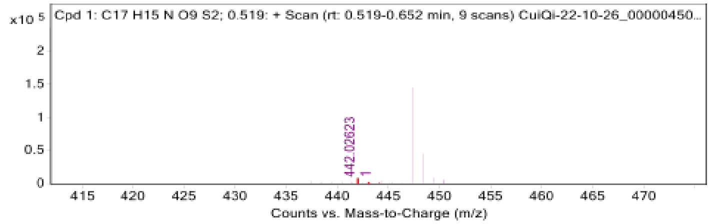

MS Spectrum Peak List

Obs. m/z	Charge	Abund	Formula	Ion/Isotope	Tgt Mass Error (ppm)
442.02623	1	6937.32	C17H15NO9S2	(M+H)+	
443.03061	1	1394.67	C17H15NO9S2	(M+H)+	
444.03369	1	887.01	C17H15NO9S2	(M+H)+	
445.03099	1	150.42	C17H15NO9S2	(M+H)+	
446.01797	1	68.31	C17H15NO9S2	(M+H)+	
442.02623	1	6937.32	C17H15NO9S2	(M+H)+	0.29
443.03061	1	1394.67	C17H15NO9S2	(M+H)+	3.46
444.03369	1	887.01	C17H15NO9S2	(M+H)+	20.15
445.03099	1	150.42	C17H15NO9S2	(M+H)+	9.04
446.01797	1	68.31	C17H15NO9S2	(M+H)+	-15.14

--- End Of Report ---

3

Compound Table

Label	Tgt Score	Mass Error (ppm)	Tgt Formula	Obs. RT	Ref. Mass	Obs. Mass
Cpd 1: C17 H14 O5 S4; 0.514	50.01	6.96	C17 H14 O5 S4	0.514	425.97241	425.97537

Obs. m/z	Obs. RT	Obs. Mass	Tgt Formula	Tgt Mass	Tgt Mass Error (ppm)	Find Cpds Algorith
426.98016	0.514	425.97537	C17 H14 O5 S4	425.97241	6.96	Find by Formula

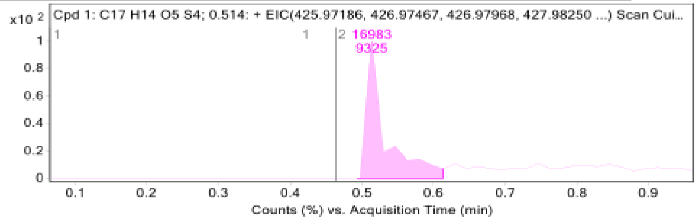

MS Zoomed Spectrum

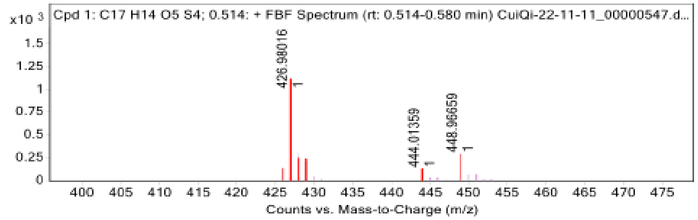

MS Zoomed Spectrum

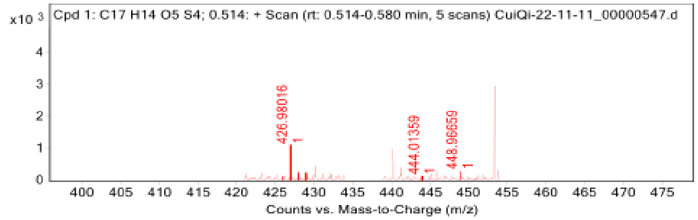

MS Spectrum Peak List

Obs. m/z	Charge	Abund	Formula	Ion/Isotope	Tgt Mass Error (ppm)
425.96242	1	130.08	C17H14O5S4	M+	
426.98016	1	1076.97	C17H14O5S4	(M+H)+	
427.99179	1	302.53	C17H14O5S4	(M+H)+	
428.98596	1	207.67	C17H14O5S4	(M+H)+	
444.01359	1	123.96	C17H14O5S4	(M+NH4)+	
448.96659	1	287.87	C17H14O5S4	(M+Na)+	
425.96242	1	130.08	C17H14O5S4	M+	-22.15
426.98016	1	1076.97	C17H14O5S4	(M+H)+	1.11
427.99179	1	302.53	C17H14O5S4	(M+H)+	21.71
428.98596	1	207.67	C17H14O5S4	(M+H)+	21.01
444.01359	1	123.96	C17H14O5S4	(M+NH4)+	16.57
448.96659	1	287.87	C17H14O5S4	(M+Na)+	11.06

--- End Of Report ---

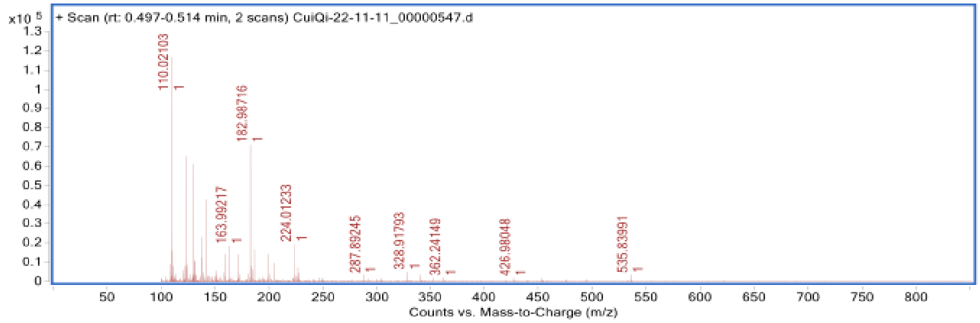

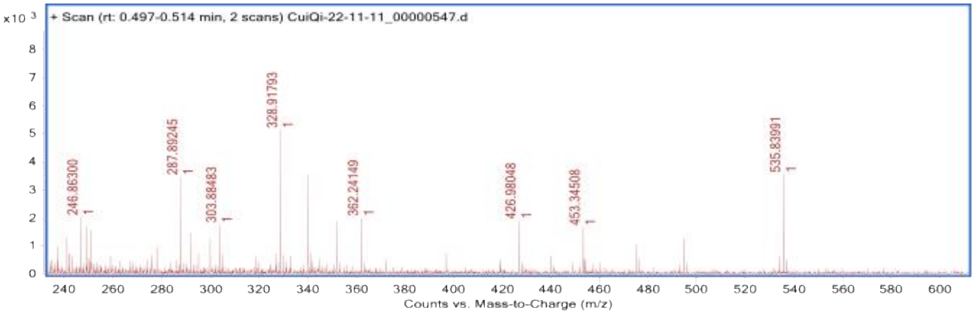

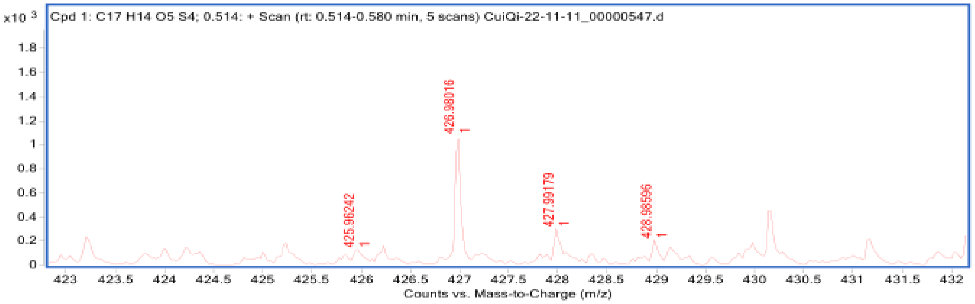

Lyso-TTS

Biomolecules

RT	Mass	Height	Area	Algorithm
9.049	14607.1139	64871	3916218	Maximum Entropy ...

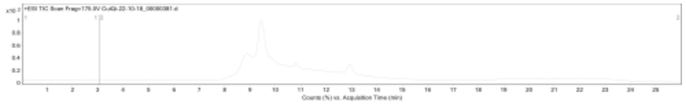

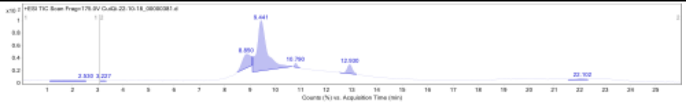

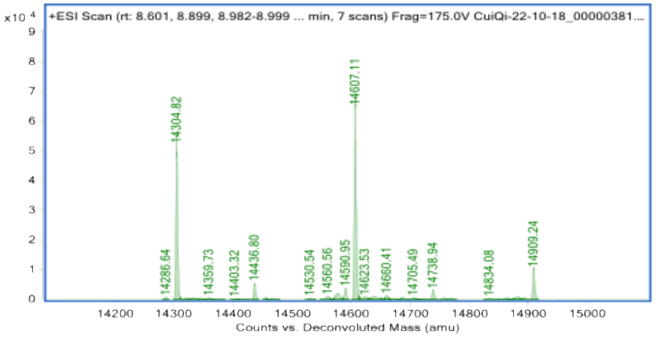

MS Spectrum

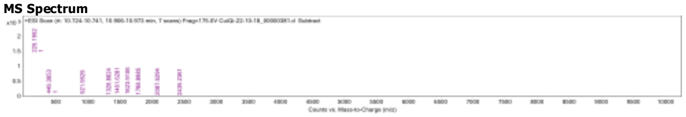

Source Biomolecule Spectrum Peak List

z	Neutral Mass	Abund
1	225.1952	148764.18
1	226.1976	20534.25
1	233.1384	6411.69
1	449.3853	6870.89
	921.9529	5012.06
	1431.3839	2772.22
	1461.6281	3908.24
	1461.9764	3609.3
	1623.9106	3840.44
	1624.2981	3120.42

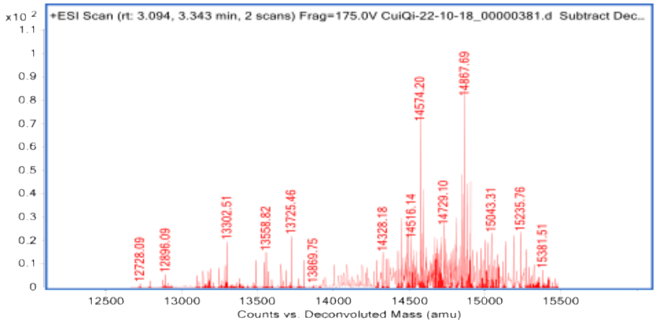

Deconvoluted Spectrum

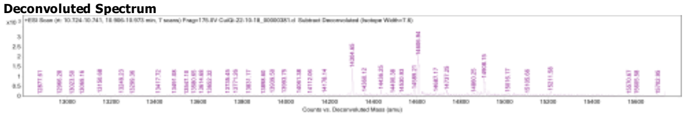

Source Biomolecule Spectrum Peak List

Neutral Mass	Abund
14304.65	1412.4
14436.25	304.47
14589.21	324.25
14606.94	2038.65
14622.98	209.92
14737.25	278.76
14877.42	243.03
14908.15	870.96
14927.86	255.99
15211.58	229.14

Overlaid Deconvolution Plot

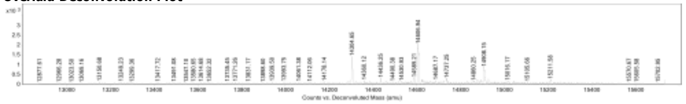

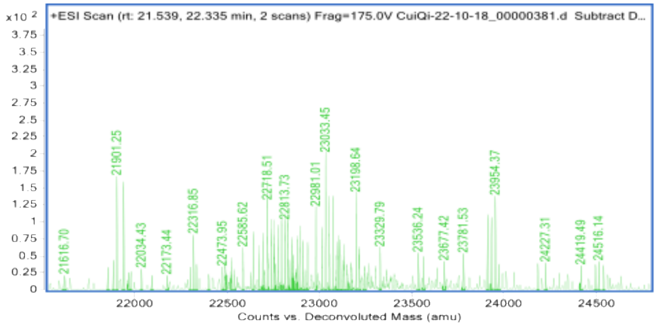

Lyso-TTS (continued)

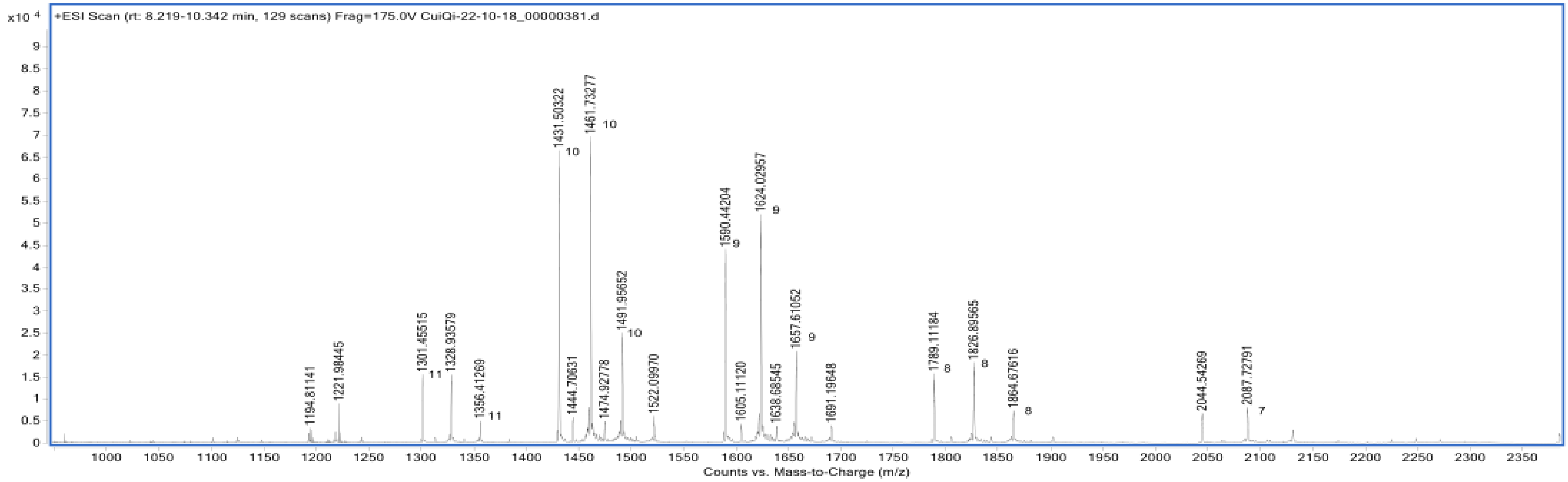

zoomed mass spectrum

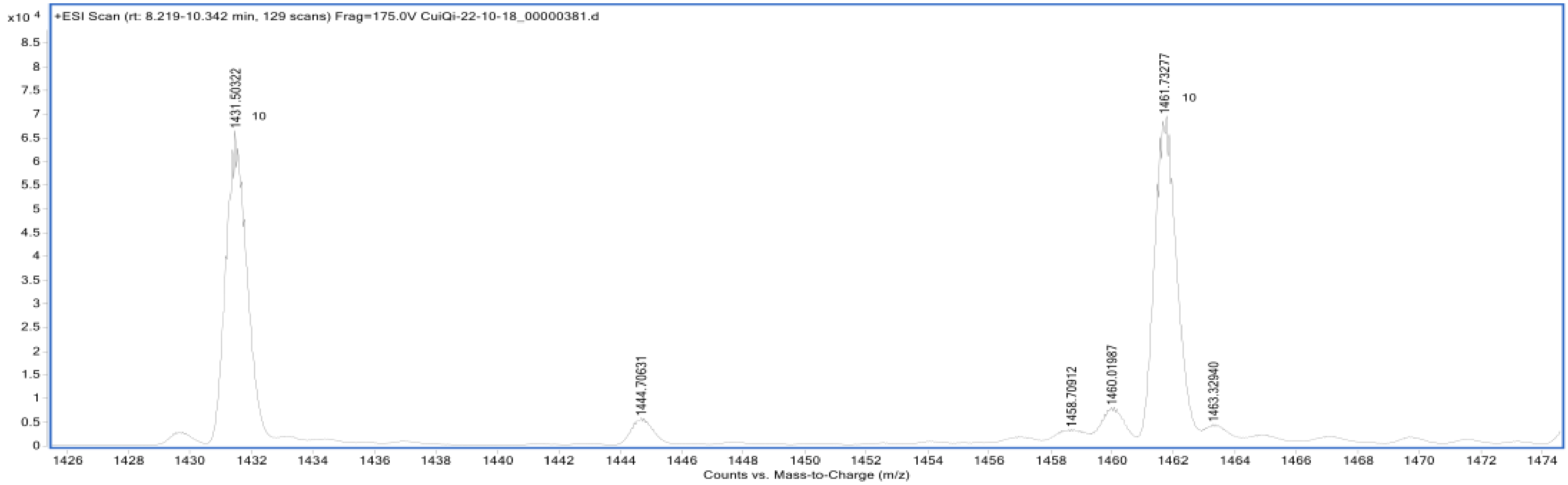

--- End Of Report ---

Lyso-TT

Biomolecules

RT	Mass	Height	Area	Algorithm
9.508	14591.2939	108756	8300403	Maximum Entropy ...

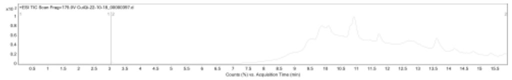

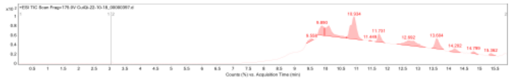

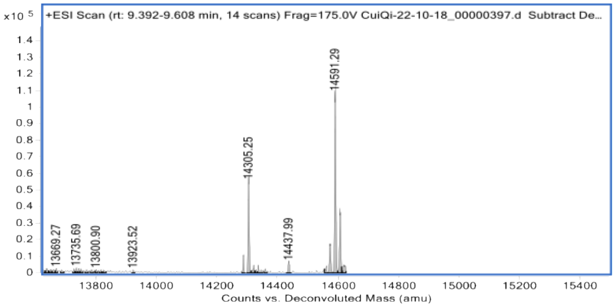

MS Spectrum

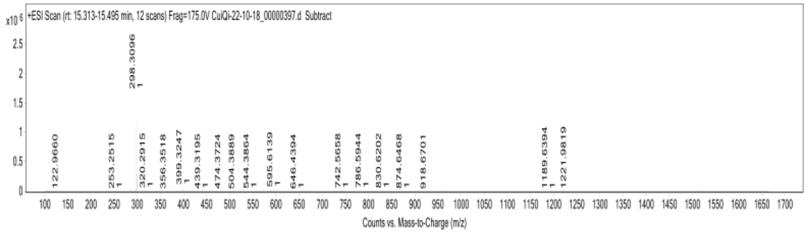

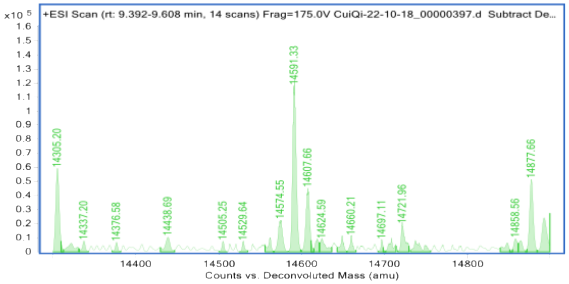

Source Biomolecule Spectrum Peak List

z	Neutral Mass	Abund
1	284.294	107755.45
1	285.2979	21668.96
1	298.3096	1743996.75
1	299.3133	386112.37
1	300.316	43297.24
1	399.3247	78550.44
1	400.3285	23635.99
1	417.3354	38762.78
1	595.6139	37719.9
1	786.5944	21159.81

Deconvoluted Spectrum

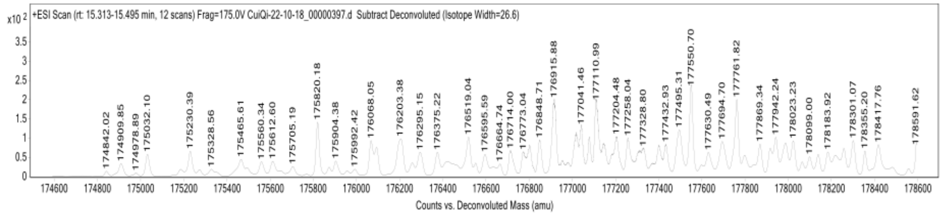

Source Biomolecule Spectrum Peak List

Neutral Mass	Abund
175820.18	140.88
176519.04	102.32
176915.88	198.16
177018.22	106.83
177041.46	128.93
177110.99	192.09
177204.48	103.07
177495.31	120.94
177550.7	239.07
177761.82	199.48

Overlaid Deconvolution Plot

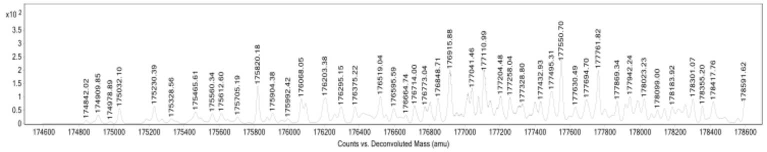

Lyso-TT (continued)

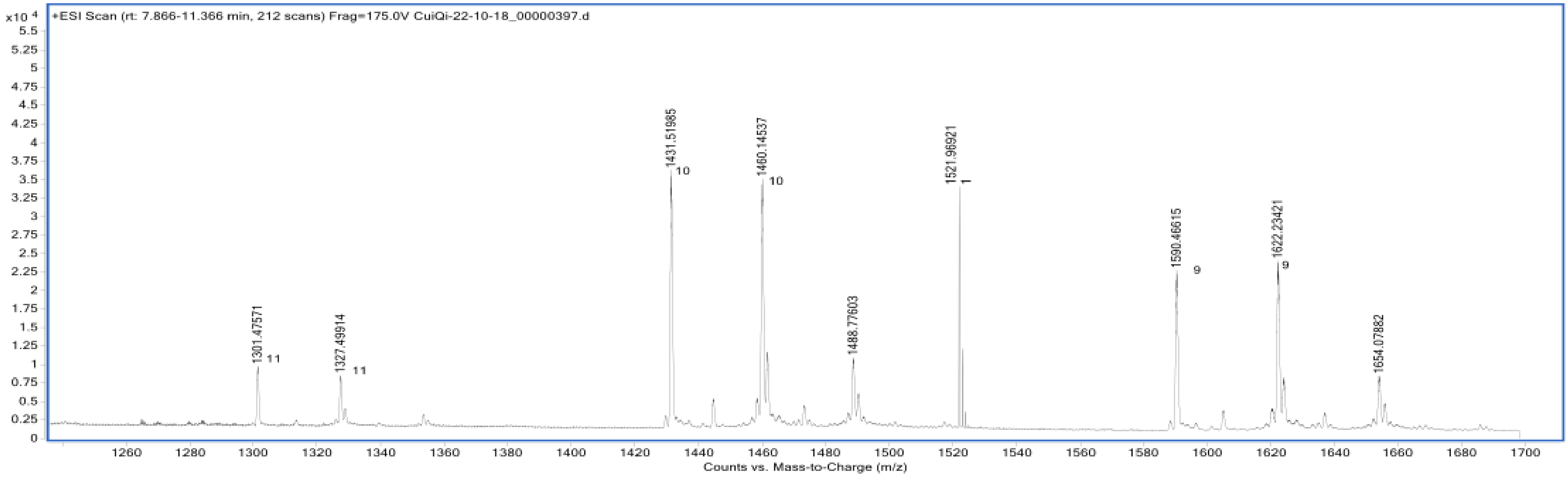

zoomed mass spectrum

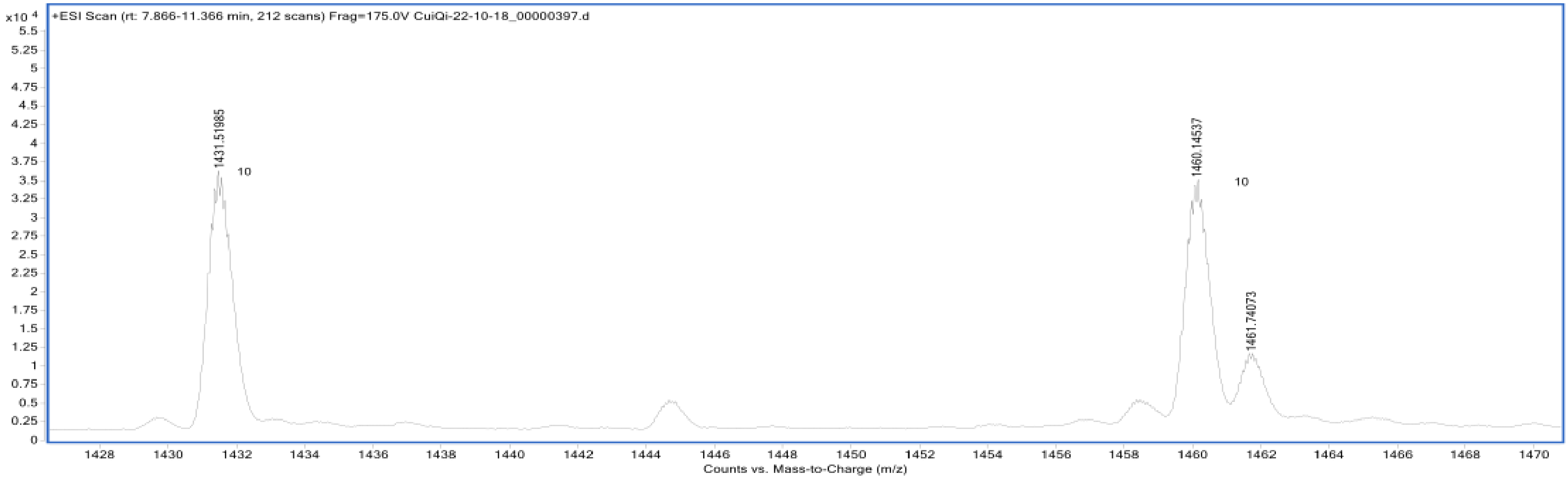

--- End Of Report ---
